# DNA methylation of individual repetitive elements in hepatitis C virus infection-induced hepatocellular carcinoma

**DOI:** 10.1186/s13148-019-0733-y

**Published:** 2019-10-21

**Authors:** Yinan Zheng, Ryan A. Hlady, Brian T. Joyce, Keith D. Robertson, Chunyan He, Drew R. Nannini, Warren A. Kibbe, Chad J. Achenbach, Robert L. Murphy, Lewis R. Roberts, Lifang Hou

**Affiliations:** 1 Center for Global Oncology, Institute for Global Health, Department of Preventive Medicine, Northwestern University Feinberg School of Medicine, 680 N. Lake Shore Drive, Suite 1400, Chicago, IL 60611-4402 USA; 2 Department of Molecular Pharmacology and Experimental Therapeutics, Mayo Clinic, Rochester, MN USA; 3 Center for Individualized Medicine, Mayo Clinic, Rochester, MN USA; 4 University of Kentucky Markey Cancer Center, Lexington, KY USA; 5 Department of Internal Medicine, Division of Medical Oncology, University of Kentucky, Lexington, KY USA; 6 Duke Cancer Institute and Duke School of Medicine, Duke University, Durham, NC USA; 7 Center for Global Health, Institute for Public Health and Medicine, Northwestern University Feinberg School of Medicine, Chicago, IL USA; 8 Division of Infectious Diseases, Department of Medicine, Feinberg School of Medicine, Northwestern University, Chicago, IL USA; 9 Robert H Lurie Comprehensive Cancer Center, Northwestern University Feinberg School of Medicine, Chicago, IL USA; 10 Division of Gastroenterology and Hepatology, Mayo Clinic, Rochester, MN USA

**Keywords:** Hepatitis C virus, Hepatocellular carcinoma, DNA methylation, Repetitive element

## Abstract

**Background:**

The two most common repetitive elements (REs) in humans, long interspersed nuclear element-1 (LINE-1) and Alu element (Alu), have been linked to various cancers. Hepatitis C virus (HCV) may cause hepatocellular carcinoma (HCC) by suppressing host defenses, through DNA methylation that controls the mobilization of REs. We aimed to investigate the role of RE methylation in HCV-induced HCC (HCV-HCC).

**Results:**

We studied methylation of over 30,000 locus-specific REs across the genome in HCC, cirrhotic, and healthy liver tissues obtained by surgical resection. Relative to normal liver tissue, we observed the largest number of differentially methylated REs in HCV-HCC followed by alcohol-induced HCC (EtOH-HCC). After excluding EtOH-HCC-associated RE methylation (FDR < 0.001) and those unable to be validated in The Cancer Genome Atlas (TCGA), we identified 13 hypomethylated REs (11 LINE-1 and 2 Alu) and 2 hypermethylated REs (1 LINE-1 and 1 Alu) in HCV-HCC (FDR < 0.001). A majority of these REs were located in non-coding regions, preferentially enriched with chromatin repressive marks H3K27me3, and positively associated with gene expression (median correlation *r* = 0.32 across REs). We further constructed an HCV-HCC RE methylation score that distinguished HCV-HCC (lowest score), HCV-cirrhosis, and normal liver (highest score) in a dose-responsive manner (*p* for trend < 0.001). HCV-cirrhosis had a lower score than EtOH-cirrhosis (*p* = 0.038) and HCV-HCC had a lower score than EtOH-HCC in TCGA (*p* = 0.024).

**Conclusions:**

Our findings indicate that HCV infection is associated with loss of DNA methylation in specific REs, which could implicate molecular mechanisms in liver cancer development. If our findings are validated in larger sample sizes, methylation of these REs may be useful as an early detection biomarker for HCV-HCC and/or a target for prevention of HCC in HCV-positive individuals.

**Electronic supplementary material:**

The online version of this article (10.1186/s13148-019-0733-y) contains supplementary material, which is available to authorized users.

## Introduction

Hepatocellular carcinoma (HCC) is the most frequent primary liver malignancy and a leading cause of cancer-related death, with 746,000 deaths worldwide in 2012 [1, 2]. In the USA, death rates from HCC increased by 43% from 2000 to 2016 [3] with only a 17.4% 5-year survival rate [4]. HCC rates are driven largely by infection with the hepatitis C virus (HCV) in much of the western world [5]. From 2010 to 2016, new HCV infections tripled in the USA [6] and HCC diagnoses increased accordingly by 4.5% [7]. Identifying molecular markers of HCV infection may not only help understand hepatocarcinogenesis in patients with chronic HCV infection, but lead to the development of HCV and/or HCC screening tools and therapeutic strategies.

Repetitive elements (REs), including long interspersed element-1 (LINE-1) and Alu element (Alu), activate oncogenic pathways in HCC [8]. LINE-1 and Alu represent the two most abundant types of RE sequences that can mobilize in the human genome [9]. Their unfettered mobility can cause genetic instability as they copy and paste themselves to new locations [10, 11], leading to diseases including cancer [12, 13]. LINE-1 and Alu can profoundly alter DNA structure and gene expression [14] by introducing alternative splice sites and exon skipping [15]. Intronic insertions of LINE-1 have been associated with mRNA destabilization, resulting in reduced expression [16]. Additionally, insertions of Alu into the 5′ and 3′ regions of genes can potentially alter their expression by altering mRNA stability [17]. In HCC, including HCV-induced cases, LINE-1 and Alu mobilization have been found to be a crucial etiological factor in HCC through their activation of oncogenic pathways [8]. Accumulating observations also imply interactions between HCV and RE mobilization: HCV may activate RE activity via interferon suppression [18, 19] and HCV could be reverse transcribed by RE activity [20].

DNA methylation is a key regulatory mechanism of RE mobilization, helping maintain genomic integrity [21, 22]. Hypomethylation of REs removes obstacles to mobilization, and this reactivation of REs (including LINE-1 and Alu) is frequently observed in HCC patients [23]. A recent study showed that global average LINE-1 methylation was lower in HCV-positive than HCV-negative cases [24]. While this suggests distinct patterns of RE methylation by HCV status, the current standard of averaging the methylation of REs across the genome offers a “bird’s eye view” of global methylomic status that may nonetheless sacrifice significant biological information [25], since specific REs can vary in their methylation statuses and play distinct roles in cancer development [26–28].

We recently developed a novel algorithm, *REMP* [25], to overcome the limitations of global RE methylation measurement. This enables us to, for the first time, obtain reliable methylation information at individual REs across the entire genome in liver tissue samples. In this study, we performed and validated genome-wide and functional genomic analyses to identify individual REs’ (LINE-1 and Alu) methylation markers that are sensitive to HCV-induced HCC. Additionally, we devised a score for potential use in diagnosis or therapeutic guidance that combines these RE methylation markers.

## Results

### Clinical features of liver tissue

Clinical features of the liver tissue analyzed (i.e., our samples from the University of Florida Shands Hospital (UFSH), and samples from TCGA) are summarized in Table 1. Among diseased (HCC or cirrhotic) liver samples, we observed similar features (age, sex, and tumor stage) between UFSH and TCGA data (*p* > 0.1). The HCV group was younger than the alcohol (EtOH) group, but the two groups shared similar sex distribution, largely male (80–90%).

**Table 1:** Demographic and clinical features

	UFSH (*n* = 86)					TCGA (*n* = 49)		
	HCV-HCC (n = 11)	EtOH-HCC (*n* = 11)	HCV-cirrhosis (*n* = 10)	EtOH-cirrhosis (*n* = 10)	Normal liver (*n* = 44)	HCV-HCC (*n* = 20)	EtOH-HCC (*n* = 20)	Non-cancerous Liver (*n* = 9)
Age [mean (SD)]	57.6 (7.2)	64.5 (7.5)	54.2 (5.4)	63.0 (6.8)	57.7 (16.0)	59.7 (11.2)	68. 4 (6.6)	70.3 (11.4)
*p* value^1^	*0.20*							
Sex [Freq. (%)]								
Female	2 (18.2)	2 (18.2)	2 (20.0)	1 (10.0)	19 (43.2)	4 (20.0)	3 (15.0)	5 (55.6)
Male	9 (81.8)	9 (81.8)	8 (80.0)	9 (90.0)	25 (56.8)	16 (80.0)	17 (85.0)	4 (44.4)
*p* value^1^	*0.99*							
Tumor stage [Freq. (%)]								
I	3 (27.27)	3 (27.27)	–	–	–	9 (45.0)	9 (45.0)	–
II	4 (36.36)	2 (18.18)	–	–	–	5 (25.0)	4 (20.0)	–
III	3 (27.27)	4 (36.36)	–	–	–	4 (20.0)	5 (25.0)	–
Unknown	1 (9.09)	2 (18.18)	–	–	–	2 (10.0)	2 (10.0)	–
*p* value^1^	*0.48*							

^1^*p* value was calculated using *t* test for continuous data and chi-squared test for categorical data among patients with HCV-HCC and EtOH-HCC combined

### RE methylation profiling and prediction across the genome

**Fig. 1:**
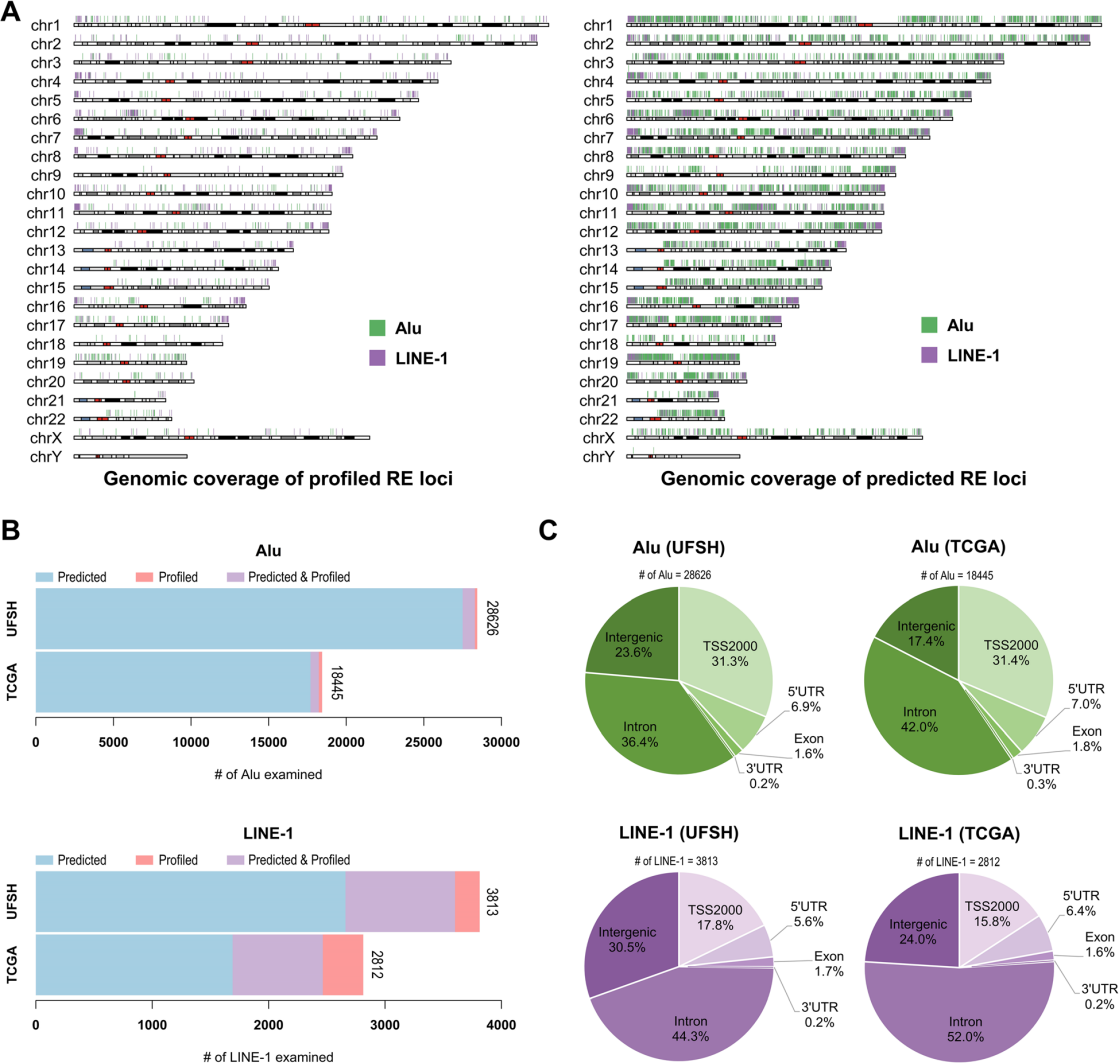
Overview of REs of interest. **a** Overview of the genomic distributions of the predicted and profiled REs examined in this study. Our predicted methylation in REs is genome-wide and substantially enhanced coverage compared to the REs profiled by the Illumina array. **b** The number of REs we reliably obtained in UFSH (28,626 Alu and 3813 LINE-1) and TCGA (18,445 Alu and 2812 LINE-1). **c** The genomic distributions of REs in UFSH and TCGA were similar, with most in either a gene intronic region or an intergenic region

Our *REMP* algorithm [25] (see the “Materials and methods” section) predicts methylation in REs throughout the genome and substantially enhances the coverage provided by REs profiled in the Illumina array (Fig. 1a). We examined 32,439 REs (3813 LINE-1 and 28,626 Alu) using UFSH data and 21,257 REs (2812 LINE-1 and 18,445 Alu) using TCGA data. Most of these examined REs were predicted by a subset of REs profiled in the Illumina array (Fig. 1b). Among this subset, predicted and profiled REs had a median correlation of ~ 0.88 in UFSH and ~ 0.95 in TCGA (Additional file 1: Figure S1), indicating reliable predictions. Consistent between UFSH and TCGA, about 60% of the Alu and about 75% of the LINE-1 repeats we examined were located in either the intronic region of a gene or an intergenic region (Fig. 1c).

### Differentially methylated REs in diseased liver induced by HCV/EtOH

**Fig. 2:**
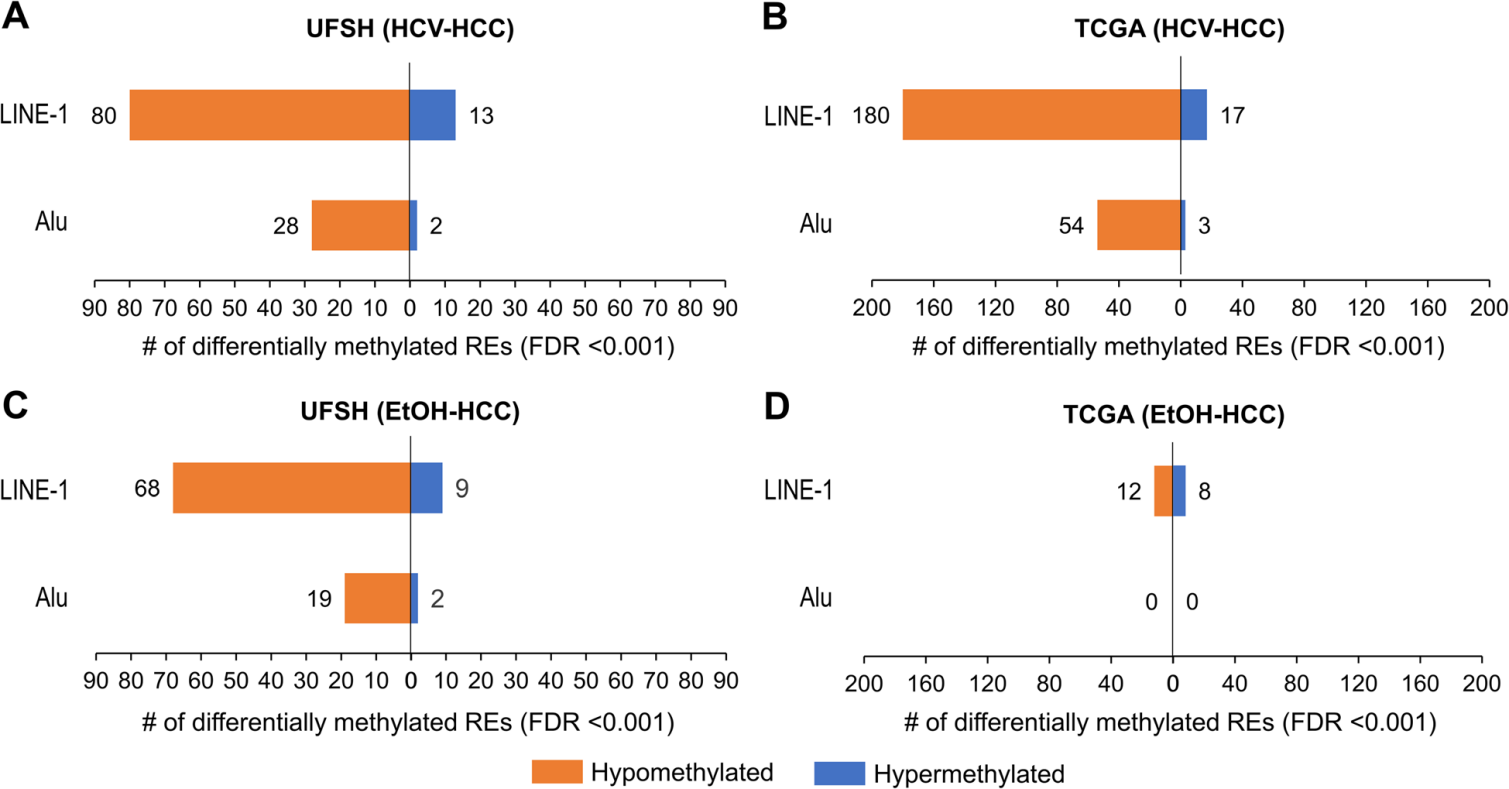
Number of differentially methylated REs in HCV/EtOH-HCC tissues, each compared to normal liver. Hypomethylated REs, in particular LINE-1, predominated among the identified dmREs (FDR < 0.001) across HCV-HCC and EtOH-HCC and both UFSH and TCGA data (**a**–**d**). In addition, we observed a greater number of dmREs in HCV-HCC compared to EtOH-HCC (**a** vs **c**), particularly in the TCGA data (**b** vs **d**). Note that the sample sizes between EtOH and HCV samples were equal in order to minimize statistical bias

We compared diseased liver tissue induced by HCV/EtOH (i.e., HCV/EtOH-HCC and HCV/EtOH-cirrhosis) with normal liver tissue to identify differentially methylated REs (dmREs), applying a stringent false discovery rate (FDR) cutoff of < 0.001. We observed 123 dmREs (93 LINE-1 and 30 Alu) in HCV-HCC in the UFSH samples (Fig. 2a) and 254 dmREs (197 LINE-1 and 57 Alu) in HCV-HCC in the TCGA samples (Fig. 2b). We observed more dmREs in the HCV liver groups than the EtOH liver groups. In UFSH, there was a total of 98 dmREs in EtOH-HCC (Fig. 2c); in TCGA, there was a total of 20 dmREs differentially methylated in EtOH-HCC (Fig. 2d). About 90% of these dmREs were hypomethylated. In contrast, we observed fewer dmREs in cirrhosis tissue in UFSH. There was a total of 10 dmREs (8 LINE-1 and 2 Alu, all hypomethylated) identified for HCV-cirrhosis (Additional file 1: Table S1), and no dmRE were identified for EtOH-cirrhosis (data available upon request).

### HCV-HCC dmREs: consistencies between UFSH and TCGA

**Fig. 3:**
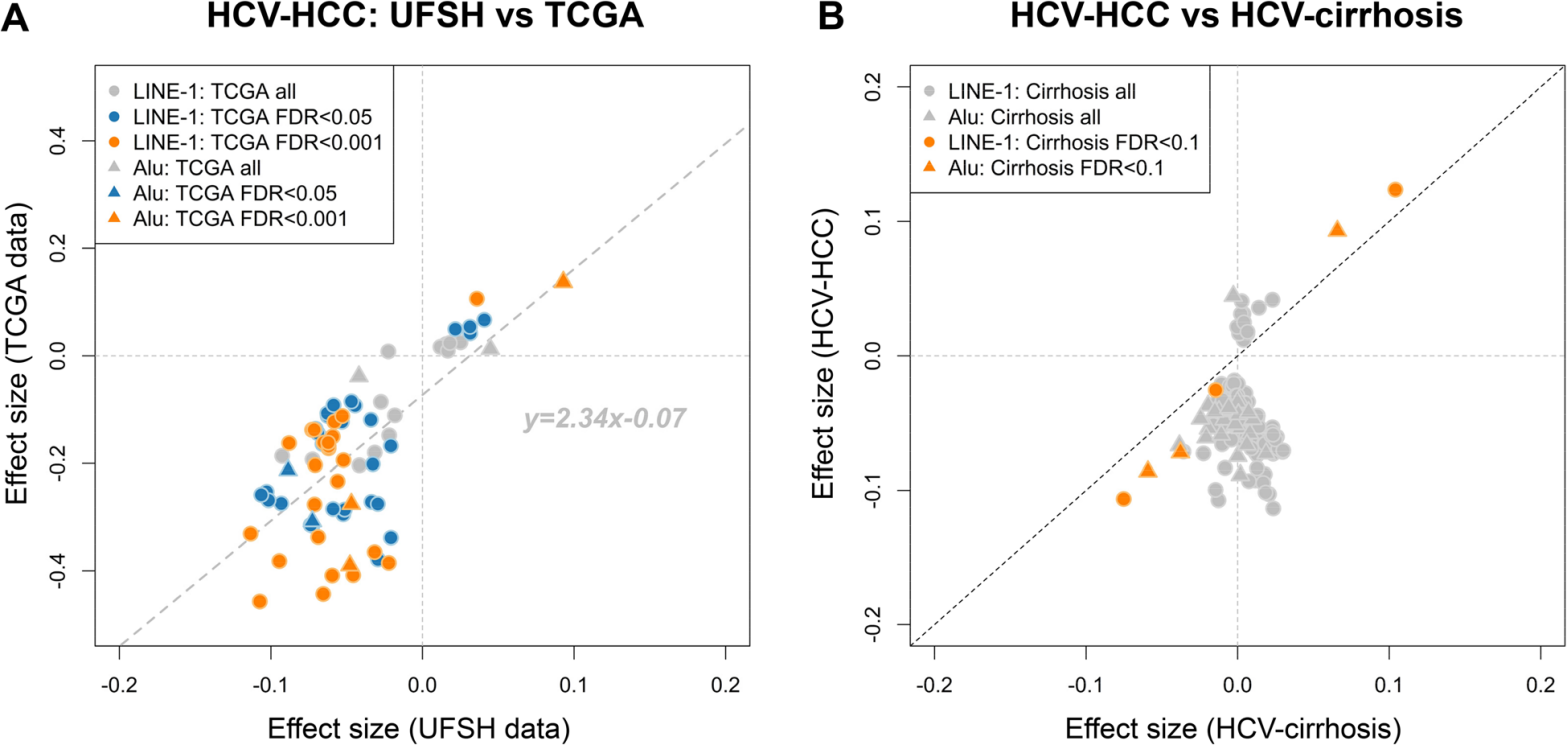
Directional consistency of the effect sizes of dmREs in HCV-HCC. **a** UFSH vs. TCGA. HCV-HCC REs were directionally consistent between UFSH and TCGA data, regardless of the significant levels in TCGA. **b** HCV-HCC (UFSH) vs. HCV-cirrhosis (UFSH). Note that among these dmREs in HCV-HCC, no RE had FDR < 0.001 in HCV-cirrhosis. However, the six (orange marks) REs that showed directional consistency were also the most significant (FDR < 0.1)

Of the 123 UFSH dmREs in HCV-HCC, a total of 76 (69 LINE-1 and 7 Alu) were available in TCGA for validation. Among these 76 REs, 24 LINE-1 (23 hypomethylated) and 3 Alu (2 hypomethylated) were validated in TCGA data (FDR < 0.001) (Additional file 1: Table S2). We observed consistent directions of association for these 76 dmREs in both datasets (*r* = 0.68, Fig. 3a).

### HCV-HCC dmREs: consistencies between HCC and cirrhosis tissue

None of the 123 dmREs in HCV-HCC reached the FDR < 0.001 threshold in HCV-cirrhosis, and we did not observe consistent directions of association between the 123 UFSH dmREs in HCV-HCC and HCV-cirrhosis (Fig. 3b). However, 6 REs (3 LINE-1 and 3 Alu) with FDR < 0.1 (nominal *p* < 0.005) showed consistent direction and magnitude of effects in both diseases (Fig. 3b, Additional file 1: Table S3).

### HCV-associated dmREs in HCC

**Fig. 4:**
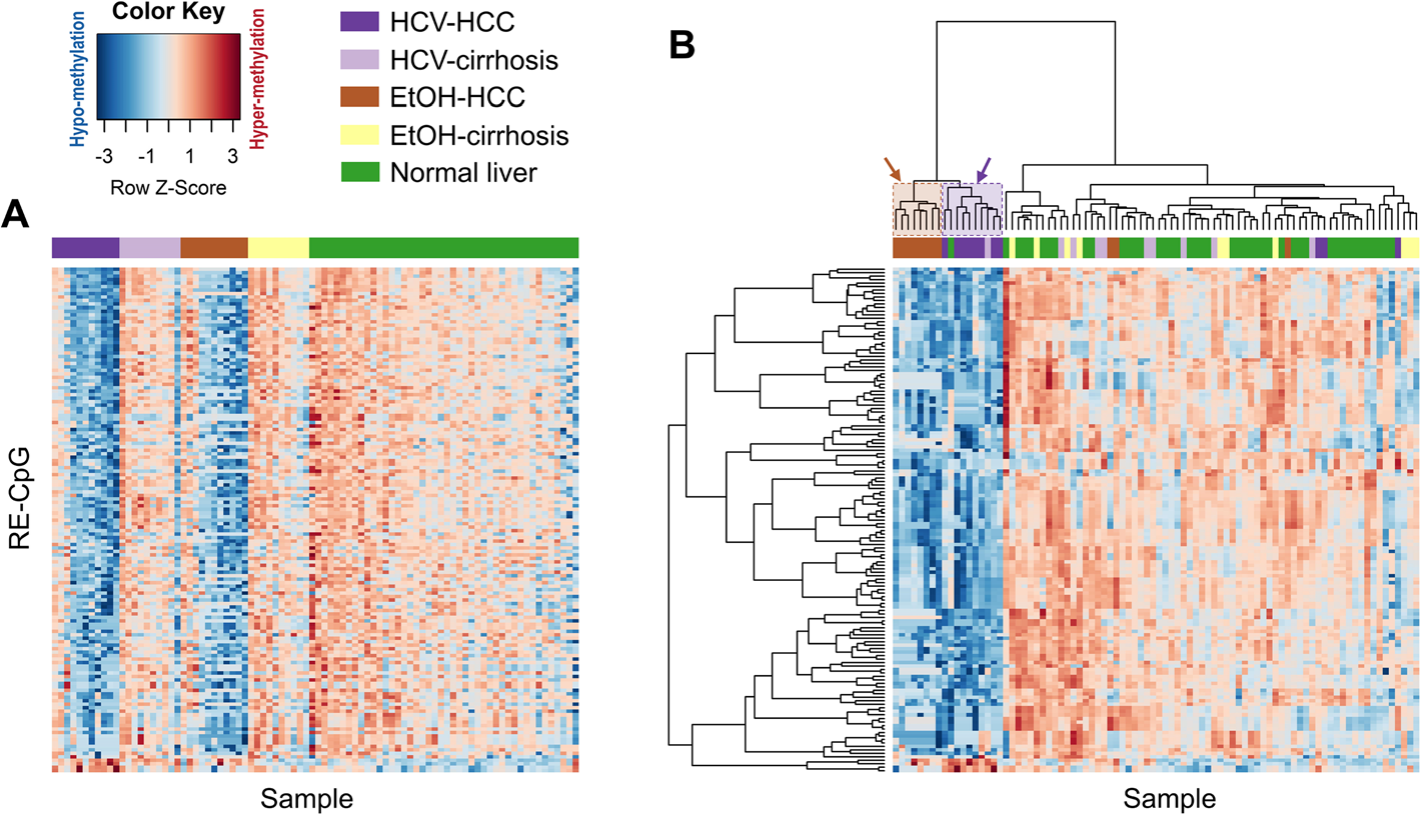
Distinct methylation patterns of HCV-HCC REs across groups. Heatmaps were generated using the methylation levels of 136 CpGs located in the 12 LINE-1 and 9 CpGs in the 3 Alu. **a** Heatmap with samples manually ordered by five groups. **b** Heatmap with samples and genes clustered by hierarchical cluster analysis using Manhattan distance and Ward’s linkage algorithm. HCC clusters were independent of cirrhosis/normal clusters. Furthermore, HCV-HCC samples (purple stripe) and EtOH-HCC samples (brown stripe) were largely clustered with each other with no misclassification as shown in the dendrogram (pointed by purple and brown arrows, respectively). Cirrhotic liver samples and normal were less distinguishable

After excluding dmREs in EtOH-HCC relative to normal liver tissue, and those not validated in TCGA, we identified 15 HCV-related dmREs including 13 hypomethylated REs (11 LINE-1 and 2 Alu) and 2 hypermethylated REs (1 LINE-1 and 1 Alu). Based on the RefSeq database, we annotated these 15 REs with 12 proximal (within 500 kbp) genes (see Additional file 2 for genomic view). Figure 4 demonstrates the distinct methylation patterns of these 15 REs in the HCV-HCC group compared to all others (HCV-cirrhosis, EtOH-cirrhosis, and normal liver) in the UFSH data. As expected, these REs were largely hypomethylated in HCV-HCC and (to a slightly lesser extent) EtOH-HCC (Fig. 4a). Our heatmap with a dendrogram (Fig. 4b) also demonstrates distinct clustering of dmREs both between HCC and cirrhosis/normal tissue, and between HCC subtypes (HCV-HCC and EtOH-HCC) (Fisher’s exact test *p* < 0.0001 for both).

### Functional analysis

**Fig. 5:**
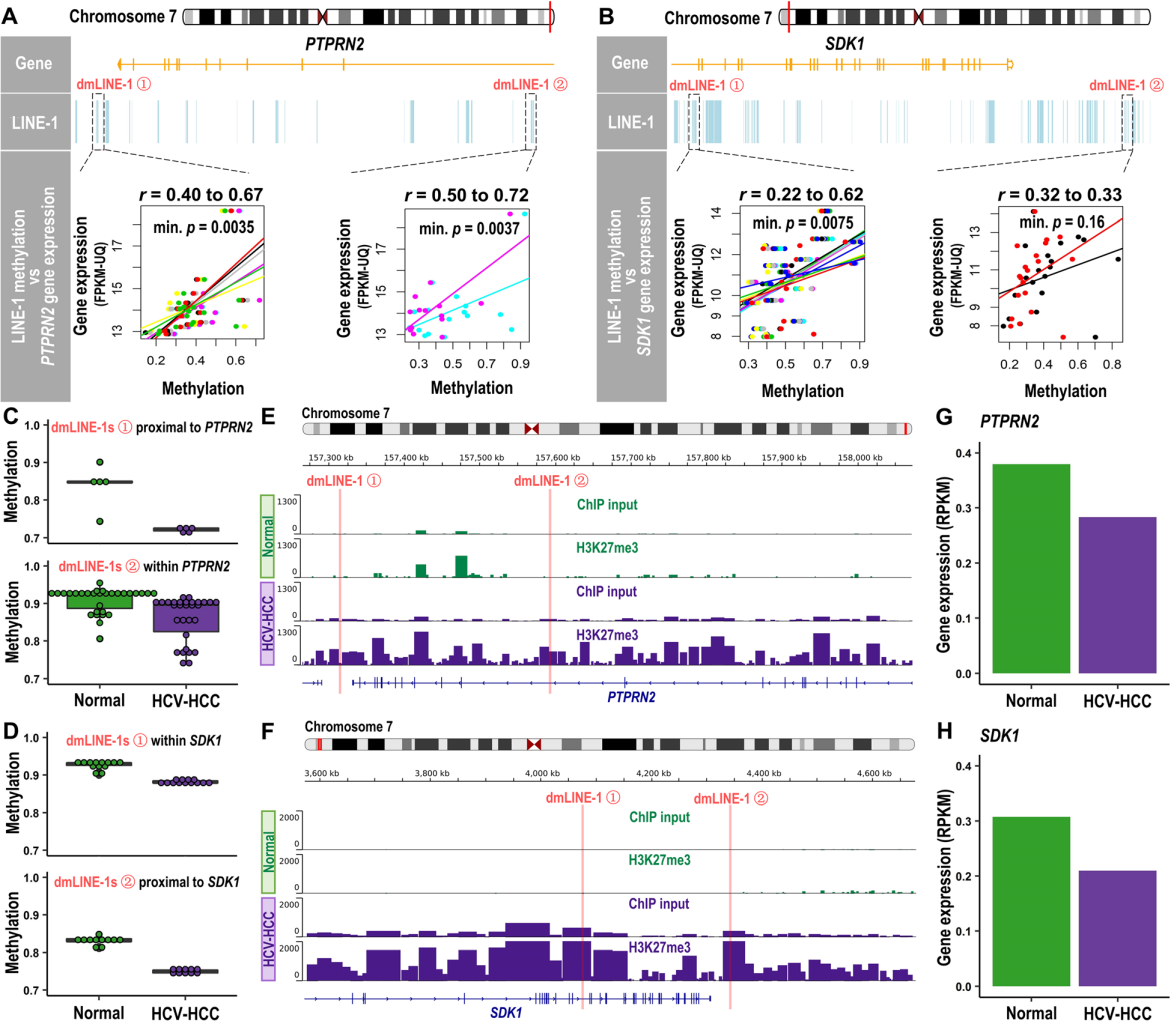
Integration of gene expression, histone modification, and RE methylation data in *PTPRN2* and *SDK1*. **a**, **b** TCGA data reveal positive correlations between the methylation of each CpG in the differentially methylated LINE-1s (dmLINE-1s) and the expression levels of their proximal genes, *PTPRN2* and *SDK1* (one color represents one CpG). Minimum *p* values of the correlation across the CpGs in dmLINE-1s are shown. **c**, **h** Using the subset of our UFSH samples, the same two dmLINE-1s proximal to genes were hypomethylated (**c**, **d**, each dot represents a CpG site in the LINE-1 loci). Repressive H3K27me3 marks were largely gained in both genes in HCC-HCV samples (**e**, **f**). Consistently, both genes were downregulated in HCV-HCC compared to normal liver samples (**g**, **h**). For each gene, average expression levels between the two selected samples are shown

As expected, given REs’ abundance in intergenic and gene intronic regions [10, 25], the 15 HCV-associated dmREs in HCC were also primarily located in these two genomic regions (Table 2). Bioinformatic analysis using the histone chromatin immunoprecipitation sequencing (ChIP-seq) data from Roadmap Epigenomics Project indicates that these 15 REs were enriched in H3K27me3 (repressive chromatin marks, *p* = 0.030) and depleted in its antagonistic mark H3K27ac (active chromatin marks, *p* = 0.037); however, we observed no enrichment patterns in either H3K4me1 or H3K4me3 (transcriptional activation marks) (Additional file 1: Table S4). Of the 12 genes annotated to these dmREs, all but 1 (*H2BFM*) had sufficiently detectable gene expression data in TCGA for functional analysis. We then combined results from three analyses: (1) dmREs, (2) correlation between methylation in dmREs and their proximal gene expression, and (3) differential gene expression between normal liver and HCV-HCC, to examine the potential regulatory roles of HCV-associated dmREs in HCC. Among the remaining 13 dmREs, 10 showed directionally consistent results throughout these 3 analyses (Table 2). Notably, 11 of the 13 REs (or 8 of the 10 directionally consistent REs) had positive correlations with their proximal genes’ expression levels (Additional file 1: Figure S2). In particular, for genes *PTPRN2* and *SDK1*, each had two differentially hypomethylated LINE-1s that are in or adjacent to the genes and positively correlated with their expression levels in HCV-HCC tissue (Fig. 5a, b). Consistently, these two genes in HCV-HCC tissue had lower gene expression levels relative to non-cancerous liver tissue in TCGA, despite the lack of statistical significance. To validate these results, we integrated our ChIP-seq data on H3K27me3 and RNA-seq data on both genes *PTPRN2* and *SDK1* from a subset of our UFSH samples. Focusing on the flanking regions of the aforementioned differentially hypomethylated LINE-1s of both genes (Fig. 5c, d), we observed that HCV-HCC tissue gained H3K27me3 marks in the genes (Fig. 5e, f) and both genes were downregulated in HCV-HCC compared to normal liver (Fig. 5g, h).

**Table 2:** Functional analysis of HCV-HCC-associated dmRE in TCGA

RE type	RE location (hg19)^a^	Proximal gene symbol	Distance to proximal gene (bp)	Genomic region	Differential methylation analysis (HCV-HCC vs normal)		RE methylation vs gene expression correlation analysis		Differential gene expression analysis (HCV-HCC vs normal)		Directionally consistent
					Mean difference	*p* value	Max. Correlation^b^	Min. *p* value^b^	log_2_ fold change	*p* value	
LINE-1	chr10:134875427–134876211	*ADGRA1*	25,296	Intergenic	− 0.331	6.97E−07	− 0.23	0.613	− 3.358	0.004	No
LINE-1	chr6:9810984–9817010	*N/A* ^c^	N/A^c^	Intergenic	− 0.203	1.23E−06	N/A^c^	N/A^c^	N/A^c^	N/A^c^	N/A^c^
LINE-1	chr7:4388884–4389235	*SDK1*	80,252	Intergenic	− 0.382	1.08E−07	0.62	0.008	− 0.018	0.980	Yes
LINE-1	chr7:4085177–4085587	*SDK1*	0	Intronic	− 0.365	3.29E−06	0.33	0.161	− 0.018	0.980	Yes
LINE-1	chr7:157594780–157595648	*PTPRN2*	0	Intronic	− 0.385	4.40E−05	0.72	0.004	− 1.053	0.116	Yes
LINE-1	chr11:2499185–2499531	*KCNQ1*	0	Intronic	− 0.408	1.83E−05	0.53	0.034	1.343	0.076	No
LINE-1	chr5:8084329–8090357	*MTRR*	183,093	Intergenic	− 0.168	1.47E−05	−0.51	0.044	0.062	0.866	Yes
LINE-1	chr2:242938503–242938722	*LINC01237*	0	Intronic	− 0.337	1.18E−05	0.28	0.274	0.764	0.344	No
LINE-1	chr7:157318533–157319261	*PTPRN2*	12,488	Intergenic	− 0.443	2.19E−09	0.67	0.003	− 1.053	0.116	Yes
LINE-1	chr8:142852678–142853054	*MROH5*	335,347	Intergenic	− 0.409	2.99E−05	−0.54	0.269	0.573	0.737	Yes
LINE-1	chr8:143458657–143458848	*TSNARE1*	0	Intronic	0.107	2.73E−05	0.58	0.020	0.960	0.013	Yes
LINE-1	chr2:1166233–1166668	*SNTG2*	0	Intronic	− 0.276	1.44E−05	0.82	0.047	− 0.102	0.955	Yes
Alu	chr13:30062564–30062769	*MTUS2*	0	Intronic	− 0.275	9.85E−07	0.52	0.156	− 3.564	0.335	Yes
Alu	chrX:103294139–103294294	*H2BFM*	222	Promoter^d^	− 0.390	1.13E−06	N/A^e^	N/A^e^	N/A^e^	N/A^e^	N/A^e^
Alu	chr6:31598686–31598816	*PRRC2A*	0	Intronic	0.137	2.90E−06	0.25	0.284	1.089	0.001	Yes

^a^Ranked by statistical significance, within LINE-1 and Alu, respectively

^b^Maximum magnitude of correlation and minimum *p* values across the CpGs in the RE

^c^No proximal gene identified within 500 kbp

^d^Within 2000 bp upstream of the transcription start site

^e^Extremely low expression across samples

### Clinical utility of HCV-HCC RE methylation score

**Fig. 6:**
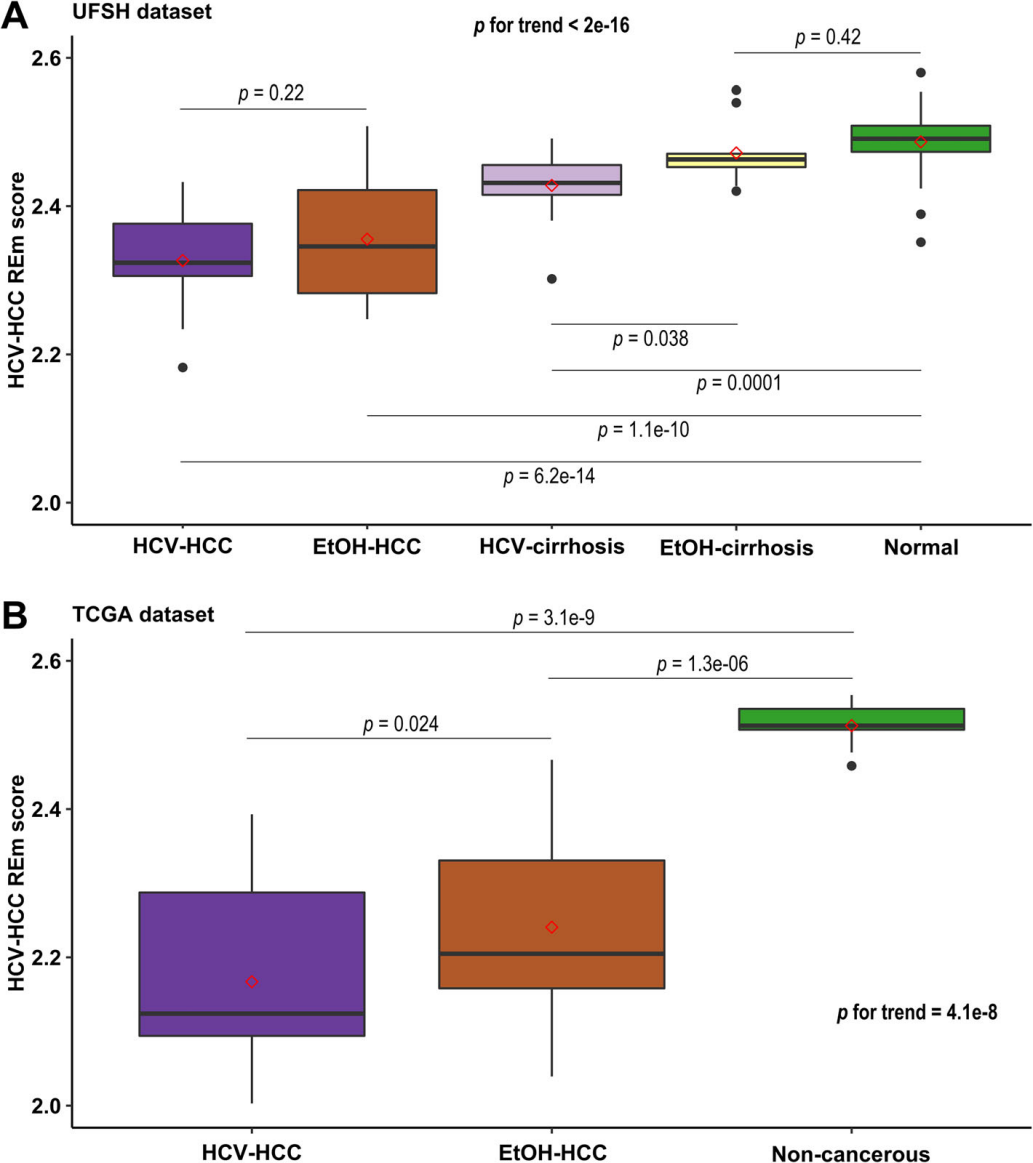
HCV-HCC RE methylation score can inform HCV-HCC diagnosis. Box plots include mean HEMS (hollow red diamond). *p* value indicates the significance of the mean HEMS differences, independent of age and sex. **a** The HEMS differed across groups in a dose-response manner HCV-HCC < EtOH-HCC < HCV-cirrhosis < EtOH-cirrhosis < normal liver. **b** Consistent findings in TCGA. In addition, HEMS was significantly lower in HCV- than in EtOH-HCC

Using methylation data of the aforementioned 15 HCV-associated dmREs in HCC, we applied penalized logistic regression to build a parsimonious model and predict HCV-HCC and normal liver status in the UFSH dataset (see the “Materials and methods” section), which selected 6 informative REs (3 LINE-1 and 3 Alu, Additional file 1: Table S5). By weighting these six elements on the magnitude of their differential methylation, we constructed an HCV-HCC RE methylation score (HEMS) and compared it in the tissue groups from both datasets. We observed strong pairwise correlations in UFSH, with HEMS being the lowest in HCV-HCC tissue liver and highest in normal liver tissue (with EtOH-HCC and cirrhosis tissue in between) (*p* for trend < 2E−16, Fig. 6a). HEMS in HCV-cirrhosis was significantly lower than in EtOH-cirrhosis (*p* = 0.038, Fig. 6a). We observed similar pairwise correlations in TCGA, with HEMS being lowest in HCV-HCC than non-cancerous liver (*p* = 3.1 E−11) and HEMS in EtOH-HCC in between (*p* for trend = 4.1E−8, Fig. 6b). HEMS in HCV-HCC was lower than that in EtOH-HCC in TCGA (*p* = 0.024) but not significantly so in UFSH (*p* = 0.22).

## Discussion

This is the first study examining a possible biologic role of methylation in individual LINE-1 and Alu elements in HCV-infection-induced HCC. We evaluated methylation of over 30,000 LINE-1 and Alu in HCC tissue samples using our recently developed prediction algorithm “*REMP*” [25]. We found that, compared to alcoholic patients HCV-positive patients had a greater number of dmREs. We also identified LINE-1 and Alu elements associated with HCV-HCC located mainly in intronic and intergenic regions, preferentially enriched in H3K27me3 marks, and positively correlated with proximal gene expression. Finally, we assessed the potential clinical utility of these RE methylation markers via a constructed HCV-HCC RE methylation score (HEMS) capable of distinguishing HCC, cirrhotic, and healthy liver tissue as well as differentiating between alcohol- and HCV-induced mechanisms. These findings point to a potentially useful role for RE methylation in the early detection and personalized prevention of HCC and possibly other liver diseases.

We observed a greater number of dmREs in HCV-positive cases regardless of clinical outcome in both datasets. Our previous investigation of UFSH data observed more differential methylation predominantly outside of REs in EtOH-HCC relative to HCV-HCC [29]. Therefore, RE may be more susceptible to differential methylation via HCV infection. These findings further support the hypothesis that HCV and RE may interact with one another [18–20] to sequentially drive inflammation, cirrhosis, and ultimately cancer.

The overlapping RE methylation patterns in both HCV-cirrhosis (FDR < 0.1) and HCV-HCC suggest that HCV acts on the same REs to drive cancer development. We observed an overall smaller effect size in HCV-cirrhosis relative to HCV-HCC and consistent direction of methylation for 6 REs. Some proximal genes targeted by these 6 REs (*FSCN1*, *GSTP1*, *JAM3*, *CHRNA6*, *NFAT5*, and *PRRC2A*) are related to immune response, viral infection, and/or HCC based on ontological analysis. For example, *FSCN1* is regulated by several microRNAs, including some which are in turn regulated by HCV [30]. *NFAT5* is involved in HCV propagation [31] and is a key regulator of critical pathways in HCV infection [32]. Furthermore, almost all of these overlapping REs had lower methylation relative to normal liver tissue, suggesting a role for hypomethylation specifically in the aforementioned processes toward cancer. These RE methylation markers, if confirmed in larger longitudinal studies, may serve as useful HCC early detection biomarkers and prevention targets among HCV-infected liver patients.

Based upon our methylation and expression analyses, we observed a potential functional role of hypomethylation in 12 HCV-HCC dmREs that downregulated their proximal genes (*PTPRN2*, *SDK1*, *MTRR*, *MROH5*, *TSNARE1*, *SNTG2*, *MTUS2*, and *PRRC2A*), many of which were previously associated with HCC. For example, *PTPRN2* and *SDK1* are targeted by two hypomethylated LINE-1s in HCV-HCC tissues. *PTPRN2* encodes a tyrosine phosphatase-like protein whose immature isoform, *proPTPRN2* has been overexpressed in human cancers [33]. Methylation of non-REs in *PTPRN2* has been associated with HCC risk previously [34], and it may also be indirectly associated with HCC risk via insulin-dependent diabetes mellitus [35]. *SDK1* is an androgen-responsive gene and its overexpression modulates cellular migration in prostate cancer [36]. A cross-species cancer study also suggested that *SDK1* may be located in an unstable genomic region [37], while RE methylation itself is a strong regulator of genomic stability. Interestingly, a recent study of 69 pairs of HCC and adjacent non-cancerous tissue also identified both *SDK1* and *PTPRN2* as the top candidate genes epigenetically regulated in hepatitis virus-related HCC [38]. Our findings indicate a possible role for RE methylation of key genes in liver cancer development.

Although the role of gene intronic and intergenic methylation in regulating gene expression remains elusive, the observed correlations between RE methylation and proximal gene expression suggest a potential mechanism as REs are largely located in non-coding regions, i.e., gene intronic regions and intergenic regions [10, 25]. Previous studies have consistently observed the so-called “DNA methylation paradox” [39] where methylation in the gene intronic regions positively correlates with gene expression [40, 41], consistent with most of our observations. RE methylation may be clinically relevant as it suppresses RE mobility, which in turn stabilizes local chromatin and silences cryptic transcription start sites or cryptic splice sites, resulting in higher overall transcriptional efficiency. RE methylation is evolutionally conservative and DNA methyltransferases *DNMT1*, *DNMT1A*, and *DNMT1B* are dedicated to RE methylation maintenance [42, 43]. This epigenetic regulation of REs, once perturbed, may lead to significant clinical differences. Previous studies have observed relationships between hypomethylation at specific RE loci and both increases in RE transcription and changes in targeted gene expression [44, 45]. Nonetheless, our results showed that a few hypomethylated REs were correlated with higher expression level of annotated genes, e.g., LINE-1s annotated by *MTRR* and *MROH5* (Table 2). Note that both of these LINE-1s were relatively far away from their annotated genes (> 150 kb). Therefore, a future mechanistic study is warranted to consolidate the biological connections between RE methylation with consideration of the characteristics of REs (e.g., location, distance from the gene) and explore potential regulatory mechanisms including RE insertions, alternative splicing, and RE exonization.

We tested the potential clinical utility of the identified RE methylation markers by HEMS using our data on HCC tumor and normal liver tissue. The average methylation in Alu and LINEs has been widely used as surrogate for global methylation [46] but its clinical value is limited due to the loss of locus-specific information. HEMS is a weighted sum of locus-specific RE methylation sites tailored and relevant to HCV-HCC. HEMS was associated with the proximal cause of hepatic malignancy, i.e., HCC < cirrhotic liver < normal liver. Moreover, HEMS was lower in HCV- than in EtOH-associated diseased liver, especially cirrhotic liver. Therefore, HEMS serves as a potentially useful diagnostic tool in detecting HCV-related liver diseases. Further sensitivity/specificity studies with larger sample sizes and more risk factors are warranted to confirm HEMS as biomarkers of HCV-HCC.

This study is subject to limitations. The current study’s sample size is not as large as our previous study on the same population, mainly due to more stringent methylation data preprocessing. Because of the inherent higher measurement error in profiling methylation in repeat sequences, the RE methylation prediction also tends to amplify the error, yielding a less reliable prediction. Therefore, the disadvantages of incorporating these samples could outweigh the advantages in sample size gain. However, our validation analysis shows highly consistent results that support the robustness of the predicted data in two independent datasets. Additionally, different populations and different quality in methylation data can lead to different coverage of predicted RE loci, potentially explaining why 40% of the RE loci we examined in UFSH data were not predicted in TCGA data. Nonetheless, we only considered those REs robustly predicted in TCGA for validation to enhance the validity and generalizability of our findings while sacrificing some potentially informative RE methylation loci identified in UFSH data. Finally, as our analyses are effectively cross-sectional in nature, the possibility of reverse causality for our findings should be considered. For example, the differences of HEMS between HCV- and EtOH-HCC were smaller than that between HCV- and EtOH-cirrhosis; this may reflect the design of the score rather than mechanistic pathways. Moreover, the limited number of overlapping RE loci between HCC and cirrhotic tissue suggests that additional genes and biological pathways are involved in cancer initiation in cirrhotic tissue. This may likewise reflect different epigenetic changes taking place after disease development, rather than mechanistic changes preceding it.

## Conclusions

In summary, our findings indicate that HCV infection has an impact on the loss of DNA methylation in certain REs, particularly LINE-1. Studies of individual RE methylation in specific genomic loci may provide additional biological information for understanding non-coding DNA epigenetics in viral carcinogenesis, and for developing novel diagnostic and therapeutic tools. If our findings are validated in larger studies, future research should explore these potential applications of RE methylation including the use of bioinformatic tools such as *REMP* to predict locus-specific RE methylation and studies of RE methylation in additional cancer types (e.g., cervical cancer).

## Materials and methods

### Patients, tissue acquisition, and DNA extraction

Patient inclusion criteria, tissue acquisition, DNA extraction, and methylation profiling have been described previously [29]. Briefly, cirrhotic and HCC tissue samples were obtained by surgical resection at the University of Florida Shands Hospital (UFSH). Healthy livers were obtained from patients undergoing surgery for colorectal carcinoma metastases to the liver or benign liver lesions. Out of 289 samples, we considered a subset of 138 relevant to current study: 53 normal liver tissue samples, 13 HCC samples induced by HCV infection (HCV-HCC), 14 HCC samples induced by alcoholism (EtOH-HCC), 39 cirrhotic liver samples induced by HCV (HCV-cirrhosis), and 19 cirrhotic liver samples induced by alcoholism (EtOH-cirrhosis). Further exclusion of samples was done in the downstream methylation data preprocessing step. Tissues were snap-frozen and stored at − 135 °C. The tissue collection protocol was approved by the Institutional Review Board and patient consent. Genomic DNA was isolated and quality-checked by standard protocols prior to bisulfite treatment using the EZ DNA Methylation Kit (Zymo, Irvine, CA) and hybridized to the Infinium 450 k HumanMethylation BeadChip (Illumina, San Diego, CA) according to manufacturer specifications.

### Methylation data preprocessing

To ensure data quality and comparability for RE methylation prediction, we applied a stringent preprocessing pipeline on both UFSH and TCGA data (Additional file 1). The final methylation working dataset contains 480,426 CpG probes and 86 samples (44 normal, 11 HCV-HCC, 11 EtOH-HCC, 10 HCV-cirrhosis, and 10 EtOH-cirrhosis). In TCGA, we downloaded the raw IDAT data files from 20 HCV-HCC tissues (risk factor annotated as “hepatitis C” only), 20 EtOH-HCC (risk factor annotated as “alcohol consumption” only), so that they are comparable to our HCC samples in UFSH data. We included nine non-cancerous liver tissues with no-or-minor cirrhosis (Ishak fibrosis score ≤ 2), which were from independent individuals different from the HCC groups.

### Prediction of methylation levels in individual REs

We applied our previously developed machine learning algorithm, *REMP* [25] to compute methylation of CpGs located in REs by taking advantage of the preprocessed methylation data described above. Briefly, the algorithm learns *cis*-correlation patterns of the CpGs in RE regions and their neighboring CpGs (< 1000 bp away) profiled by the Illumina array platform to carry out predictions on the un-profiled RE regions. Meanwhile, it evaluates the reliability of the prediction so that only REs with two or more CpGs reliably predicted or profiled are retained. With these REs, we then took the mean methylation levels of their predicted or profiled CpGs, representing their individual REs’ methylation levels, as primary data for analyses. Methylation levels of these CpGs in REs were used as secondary data to further confirm findings.

### Analysis of dmREs in HCV-HCC

For both UFSH and TCGA data, we applied *limma* (linear models for microarray data) [47] to identify candidate REs differentially methylated between the diseased liver tissue induced by HCV (i.e., HCV-HCC and HCV-cirrhosis) and normal liver tissue. This process was repeated in comparison between diseased liver tissue induced by alcohol consumption (i.e., EtOH-HCC and EtOH-cirrhosis) and normal liver tissue. HCV-HCC vs. normal liver and EtOH-HCC vs. normal liver comparisons were repeated in TCGA data. These regression models were adjusted for age and sex. Additionally, to account for experimental batch effects and other technical biases, we derived surrogate variables from intensity data for non-negative internal control probes using principal components (PCs) analysis [48]. For both our data and TCGA data, the top four PCs explained > 95% of the variation across the non-negative internal control probes and thus were included in the model. We used Benjamini-Hochberg adjusted FDR to account for multiple testing. To better control false-positive findings, we considered a stringent FDR cutoff of < 0.001 statistically significant and differentially methylated.

To evaluate the *directional consistency* (i.e., consistently hypomethylated or hypermethylated) of the dmREs in HCV-HCC, we further compared effect sizes (i.e., adjusted mean methylation differences in diseased liver compared to normal liver) of dmREs in HCV-HCC with the effect sizes of the same REs in either HCV-HCC in TCGA data or in HCV-cirrhosis in UFSH data.

To validate the dmREs and ensure that they were associated with HCV-HCC, our final list of HCV-HCC REs for further analyses included only those that (1) were validated in TCGA (i.e., FDR < 0.001), and (2) did not overlap with dmREs associated with EtOH-HCC in either UFSH or TCGA data.

### Functional analysis

We used TCGA methylation data and RNA-seq data to understand whether the identified HCV-HCC-associated REs with differential methylation levels affect their targeting or proximal gene expression (within 500 kbp of the REs). Considering that our statistical power may be constrained due to limited sample size, we further evaluated directional consistency by accounting for all three analyses: differential methylation analysis (UFSH data validated by TCGA data), methylation-expression correlation analysis (TCGA data), and differential gene expression analysis (TCGA data). For example, if an RE is hypomethylated in tumor tissue, it is *directionally consistent* if that RE is positively/negatively correlated with its targeting or proximal gene expression and the gene is downregulated/upregulated in tumor tissue. To test for functional enrichment of identified HCV-HCC REs, we conducted permutation-based enrichment analyses of four histone modification marks (H3K4me1, H3K4me3, H3K27me3, and H3K27ac) [49] in normal liver tissue derived from the Roadmap Epigenomics Project [50] (Additional file 1). We also randomly selected a subset of the UFSH samples for RNA-seq in 2 HCV-HCC and 2 normal liver samples. One of the HCV-HCC and one of the normal liver samples also had ChIP-seq data available to confirm the findings of prioritized gene(s) and histone mark(s). The RNA-seq and ChIP-seq were performed as previously described [51].

### HCV-HCC RE methylation score

We aimed to develop an RE methylation score that can be used to inform HCV-associated HCC/cirrhotic liver (Additional file 1). We investigated whether the score differed by HCC, cirrhotic liver, and normal liver. We also applied the formula to EtOH-related samples from UFSH and TCGA to evaluate the fidelity of the score in HCV infection. We compared the mean score across groups using multiple linear regression adjusted for age and sex.

## Additional files

Additional file 1: Figure S1. High correlation between the profiled and predicted LINE-1/Alu methylation. Figure S2. Scatter plot of HCV-HCC associated RE methylation and proximal gene expression. Table S1. Differentially hypomethylated REs in HCV-cirrhosis (FDR < 0.001). Table S2. Differentially methylated REs in HCV-HCC using UFSH data and validation in TCGA (76 REs: 69 LINE-1 + 7 Alu). Table S3. Differentially methylated LINE-1 and Alu in HCV-HCC (FDR < 0.001) that were directionally consistent in HCV-cirrhosis. Table S4. Enrichment of the 15 HCV-HCC REs in four regulatory histone modification marks measured in normal liver tissue (ID E066) in Roadmap Epigenomics Project. Table S5. Coefficients of HCV-HCC RE methylation score^1.^ (DOCX 920 kb)

Additional file 2: Extended figures. (PDF 7145 kb)

## Data Availability

The repetitive element methylation datasets generated during and/or analyzed during the current study are available from the corresponding author on reasonable request.

## Abbreviations

Alu: Alu element
ChIP-seq: Chromatin immunoprecipitation sequencing
dmRE: Differentially methylated RE
EtOH: Alcohol
FDR: False discovery rate
H3K27ac: Histone H3 lysine 27 acetylation
H3K27me3: Histone H3 lysine 27 tri-methylation
H3K4me1: Histone H3 lysine 4 mono-methylation
H3K4me3: Histone H3 lysine 4 tri-methylation
HCC: Hepatocellular carcinoma
HCV: Hepatitis C virus
HEMS: HCV-HCC RE methylation score
LINE-1: Long interspersed nuclear element-1
mRNA: Messenger RNA
RE: Repetitive element
REMP: Repetitive Element Methylation Prediction
TCGA: The Cancer Genome Atlas
UFSH: University of Florida Shands Hospital
