# Supplementary material for: DNA methylation of individual repetitive elements in hepatitis C virus infection-induced hepatocellular carcinoma
Source: Clin Epigenetics. 2019 Oct 21;11:145. doi: 10.1186/s13148-019-0733-y (PMC6802191; doi:10.1186/s13148-019-0733-y)
Supplement: Supplementary file 1 — Figure S1. High correlation between the profiled and predicted LINE-1/Alu methylation. Figure S2. Scatter plot of HCV-HCC associated RE methylation and proximal gene expression. Table S1. Differentially hypomethylated REs in HCV-cirrhosis (FDR < 0.001). Table S2. Differentially methylated REs in HCV-HCC using UFSH data and validation in TCGA (76 REs: 69 LINE-1 + 7 Alu). Table S3. Differentially methylated LINE-1 and Alu in HCV-HCC (FDR < 0.001) that were directionally consistent in HCV-cirrhosis. Table S4. Enrichment of the 15 HCV-HCC REs in four regulatory histone modification marks measured in normal liver tissue (ID E066) in Roadmap Epigenomics Project. Table S5. Coefficients of HCV-HCC RE methylation score1. (DOCX 920 kb) [file 13148_2019_733_MOESM1_ESM.docx]

**SUPPLEMENTARY MATERIALS**

[SUPPLEMENTARY METHODS 2](#_Toc5891333)

[Methylation data preprocessing 2](#_Toc5891334)

[Functional analysis 2](#_Toc5891335)

[HCV-HCC RE methylation score 3](#_Toc5891336)

[SUPPLEMENTARY FIGURES 4](#_Toc5891337)

[Supplemental Figure S1. 4](#_Toc5891338)

[Supplemental Figure S2. 5](#_Toc5891339)

[SUPPLEMENTARY TABLES 6](#_Toc5891340)

[Supplemental Table S1. 6](#_Toc5891341)

[Supplemental Table S2. 7](#_Toc5891342)

[Supplemental Table S3. 9](#_Toc5891343)

[Supplemental Table S4. 10](#_Toc5891344)

[Supplemental Table S5. 11](#_Toc5891345)

[SUPPLEMENTARY REFERENCES 12](#_Toc5891346)

SUPPLEMENTARY METHODS

Methylation data preprocessing

Infinium Methylation 450k HumanMethylation BeadChip (450k array) raw data were loaded by the R package *minfi* ^1^. Quality control and data preprocessing were conducted using the R package *ENmix* ^2^. In the quality control step, low-quality methylation measurements were identified by detection p-value <10^-6^ or number of beads <3. We excluded 5,086 CpGs with a detection rate <95% and 68 samples with a percentage of low-quality methylation measurements >5% or extremely low intensity of bisulfite conversion probes (less than 3 × standard deviation of the intensity across samples below the mean intensity). We further removed 16 samples that were extreme outliers, as defined by Tukey’s method [i.e., <25th percentile – 3 * interquartile range (IQR) or >75th percentile + 3 * IQR] and based on the average total intensity value across CpG probes. The remaining samples were preprocessed using *ENmix*, including dye bias correction and quantile-normalization. Lastly, low-quality methylation values (detection p-value <10^-6^ or number of beads <3) and extreme β-value outliers across samples (defined by Tukey’s method) were set as missing. The missing values were then imputed using *k*-nearest neighbors algorithm designed for DNA microarrays ^3^.

Functional analysis

We downloaded the RNA-seq data from the same tissues for functional analysis. The data were generated by TCGA and harmonized by Genomic Data Commons (GDC)*.* We used linear regression to examine the associations between RE methylation and expression levels in proximal genes in HCV-HCC tumors; for this analysis, we used the normalized data by fragments per kilobase of transcript per million mapped reads upper quartile (FPKM-UQ). We also compared the gene expression level (using count data) in the HCV-HCC tumors and normal tissues in TCGA using R package *DEseq2* ^4^.

We tested functional enrichment of the identified HCV-HCC REs in four histone modification marks we previously studied in HCC ^5^. We used histone modification broad ChIP peaks data in normal liver tissue provided by Roadmap Epigenomic Project (ID: E066). To determine whether enrichment (or depletion) occurred more often than expected by chance, we generated 10,000 randomly selected RE sets (with replacement) of the HCV-HCC REs, each matched with the HCV-HCC REs for methylation mean (±10%) and standard deviation (±10%). For each histone modification mark, we then calculated the number of overlapping HCV-HCC REs (observed count) with the histone modification peak regions and same for the 10,000 permuted sets (expected count). By comparing the observed count and the distribution of expected count, we obtained an empirical one-sided p value for the enrichment test. If the observed count was significantly (p <0.05) greater than the mean of expected count, we determined that the HCV-HCC REs were enriched in a certain histone modification mark, otherwise depleted.

HCV-HCC RE methylation score

Using UFSH methylation data as training set, we employed logistic regression with LASSO (least absolute shrinkage and selection operator) penalty (R package *glmnet* ^6^) to further select the identified HCV-HCC specific REs by minimizing the prediction error of the cancer state (i.e. HCV-HCC vs normal). To account for the different magnitude of each RE, we standardized the RE methylation with mean of 0 and standard deviation of 1. The regularization parameter λ was tuned using 10-fold cross validation. The optimized model assigned a weight to each RE (if weight = 0, the RE is not selected). We then used these weights to construct a formula calculating a weighted sum of the RE methylation, namely HCV-HCC RE methylation score.

SUPPLEMENTARY FIGURES


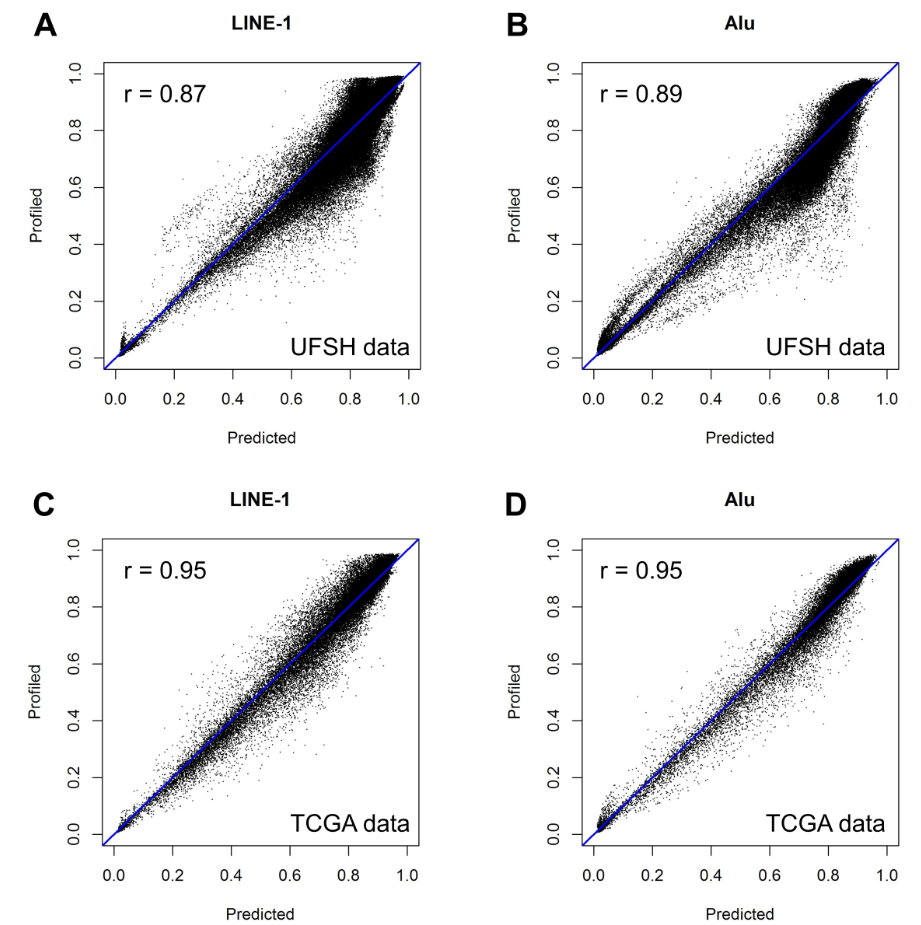


Figure S1. **High correlation between the profiled and predicted LINE-1/Alu methylation.** (A-B) UFSH data. (C-D) TCGA data. A small portion (5%) of the examined LINE-1 and Alu in this study were both profiled by 450k array and predicted by REMP, providing an opportunity to evaluate the performance of the prediction. Median correlations across samples are reported.


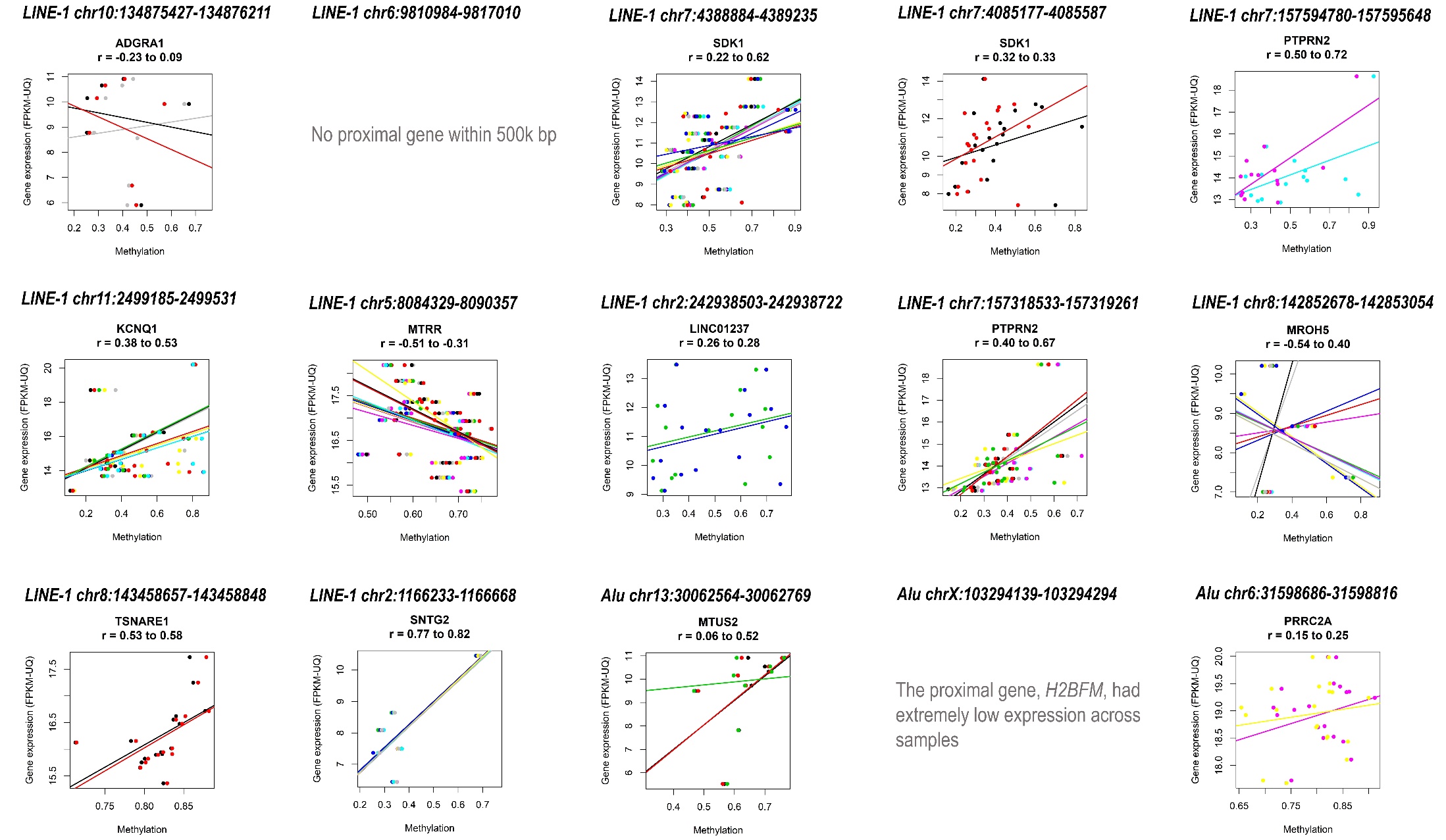


Figure S2. **Scatter plot of HCV-HCC associated RE methylation and proximal gene expression.** Correlations between the methylation of each CpGs in the RE and gene expression are indicated by various random colors (one color for one CpG in the RE).

SUPPLEMENTARY TABLES

Table S1. **Differentially hypomethylated REs in HCV-cirrhosis (FDR <0.001)**

| **RE type** | **RE location (hg19)** | **Proximal gene symbol** | **Estimate** | **t** | **p-value** | **FDR** |
| --- | --- | --- | --- | --- | --- | --- |
| LINE-1 | chr20:50156851-50156998 | *NFATC2* | -0.0753 | -8.6923 | 3.26E-11 | 1.24E-07 |
| LINE-1 | chr10:590160-590956 | *DIP2C* | -0.0539 | -6.2553 | 8.56E-08 | 0.0002 |
| LINE-1 | chr5:14414720-14414783 | *TRIO* | -0.0676 | -6.0225 | 1.98E-07 | 0.0003 |
| LINE-1 | chr8:144543384-144543983 | *ZC3H3* | -0.1040 | -5.9188 | 2.87E-07 | 0.0003 |
| LINE-1 | chr20:32266902-32267068 | *E2F1* | -0.0629 | -5.8238 | 4.28E-07 | 0.0003 |
| LINE-1 | chr16:89390695-89390838 | *ANKRD11* | -0.1681 | -5.5282 | 1.22E-06 | 0.0008 |
| LINE-1 | chr2:242701785-242702420 | *D2HGDH* | -0.1053 | -5.4742 | 1.64E-06 | 0.0009 |
| LINE-1 | chr1:2083986-2084702 | *PRKCZ* | -0.0362 | -5.3995 | 1.82E-06 | 0.0009 |
| Alu | chr19:2084891-2085022 | *MOB3A* | -0.0903 | -6.4476 | 3.34E-08 | 0.0007 |
| Alu | chr20:35273589-35273780 | *SLA2* | -0.0512 | -6.2940 | 4.85E-08 | 0.0007 |

Note: None of these 10 HCV-cirrhosis dmREs were found in HCV-HCC dmREs.

Table S2. **Differentially methylated REs in HCV-HCC using UFSH data and validation in TCGA (76 REs: 69 LINE-1 + 7 Alu)**

| **RE type** | **RE location (hg19)** | **UFSH** | | | | **TCGA** | | | | **Validated^1^** | **Directionally  consistent^2^** |
| --- | --- | --- | --- | --- | --- | --- | --- | --- | --- | --- | --- |
|  |  | **Estimate** | **t** | **p-value** | **FDR** | **Estimate** | **t** | **p-value** | **FDR** |  |  |
| LINE-1 | chr7:88180341-88186375 | -0.08804 | -6.64744 | 2.26E-08 | 3.79E-05 | -0.1624 | -5.49199 | 1.38E-05 | 0.00032 | TRUE | TRUE |
| LINE-1 | chr12:96709721-96715749 | -0.07287 | -6.5893 | 2.55E-08 | 3.79E-05 | -0.13777 | -5.85945 | 8.06E-06 | 0.000229 | TRUE | TRUE |
| LINE-1 | chr7:4174811-4175120 | -0.04166 | -6.42928 | 4.21E-08 | 3.79E-05 | -0.20339 | -1.98056 | 0.062268 | 0.108756 | FALSE | TRUE |
| LINE-1 | chr8:75506471-75512487 | -0.04453 | -6.0028 | 1.98E-07 | 0.000102 | -0.09242 | -2.92564 | 0.007594 | 0.027732 | FALSE | TRUE |
| LINE-1 | chr8:144165198-144165697 | -0.10281 | -5.82062 | 4.06E-07 | 0.000165 | -0.25268 | -3.9648 | 0.000612 | 0.005152 | FALSE | TRUE |
| LINE-1 | chr13:96978435-96984462 | -0.06199 | -5.81202 | 4.19E-07 | 0.000165 | -0.17198 | -6.62507 | 2.41E-06 | 0.000104 | TRUE | TRUE |
| LINE-1 | chr17:77657194-77658052 | -0.05231 | -5.77026 | 4.59E-07 | 0.000165 | -0.29434 | -4.0632 | 0.000479 | 0.004277 | FALSE | TRUE |
| LINE-1 | chr10:134875427-134876211 | -0.11342 | -5.68683 | 6.19E-07 | 0.000186 | -0.33061 | -6.84731 | 6.97E-07 | 4.45E-05 | TRUE | TRUE |
| LINE-1 | chr9:140614074-140614275 | 0.03143 | 5.743451 | 6.84E-07 | 0.000189 | 0.042169 | 3.081953 | 0.005441 | 0.022334 | FALSE | TRUE |
| LINE-1 | chr8:1798776-1799346 | -0.01825 | -5.60625 | 8.27E-07 | 0.000193 | -0.1108 | -2.51594 | 0.020053 | 0.050922 | FALSE | TRUE |
| LINE-1 | chr1:3089083-3089530 | -0.0514 | -5.5803 | 9.07E-07 | 0.000193 | -0.28576 | -3.13488 | 0.004807 | 0.02073 | FALSE | TRUE |
| LINE-1 | chr13:41309056-41315081 | -0.07152 | -5.55117 | 1.01E-06 | 0.000193 | -0.1376 | -5.97473 | 1.17E-05 | 0.000283 | TRUE | TRUE |
| LINE-1 | chr7:3996663-3997134 | -0.10735 | -5.53052 | 1.08E-06 | 0.000195 | -0.45684 | -6.25368 | 3.31E-06 | 0.000129 | TRUE | TRUE |
| LINE-1 | chr11:2209797-2210445 | -0.09265 | -5.53835 | 1.24E-06 | 0.000204 | -0.18604 | -2.02361 | 0.056545 | 0.102982 | FALSE | TRUE |
| LINE-1 | chr6:9810984-9817010 | -0.07086 | -5.53607 | 1.25E-06 | 0.000204 | -0.20302 | -6.59635 | 1.23E-06 | 7.18E-05 | TRUE | TRUE |
| LINE-1 | chr2:157111031-157117044 | -0.05818 | -5.45176 | 1.51E-06 | 0.000219 | -0.12246 | -6.08256 | 3.75E-06 | 0.000138 | TRUE | TRUE |
| LINE-1 | chr7:4388884-4389235 | -0.09447 | -5.46378 | 1.52E-06 | 0.000219 | -0.38177 | -7.66034 | 1.08E-07 | 1.71E-05 | TRUE | TRUE |
| LINE-1 | chr1:1906735-1906976 | -0.02088 | -5.42283 | 1.59E-06 | 0.00022 | -0.1675 | -2.64403 | 0.015157 | 0.042579 | FALSE | TRUE |
| LINE-1 | chr12:48713977-48719958 | -0.06258 | -5.40756 | 1.68E-06 | 0.000224 | -0.1119 | -3.92054 | 0.000683 | 0.005599 | FALSE | TRUE |
| LINE-1 | chr11:2563675-2564506 | -0.02749 | -5.30512 | 2.41E-06 | 0.000288 | -0.0857 | -0.85442 | 0.402495 | 0.475553 | FALSE | TRUE |
| LINE-1 | chr4:5927882-5928352 | -0.0727 | -5.30442 | 2.42E-06 | 0.000288 | -0.31561 | -4.36695 | 0.000245 | 0.002572 | FALSE | TRUE |
| LINE-1 | chr4:95756910-95762936 | -0.0469 | -5.26309 | 2.80E-06 | 0.000315 | -0.08485 | -3.39646 | 0.002857 | 0.01548 | FALSE | TRUE |
| LINE-1 | chr11:1143423-1143898 | -0.06599 | -5.22221 | 3.24E-06 | 0.000337 | -0.16418 | -3.70595 | 0.001161 | 0.008162 | FALSE | TRUE |
| LINE-1 | chr19:4484405-4484499 | 0.015445 | 5.218988 | 3.27E-06 | 0.000337 | 0.021757 | 1.933738 | 0.065518 | 0.113097 | FALSE | TRUE |
| LINE-1 | chr7:4085177-4085587 | -0.0316 | -5.19641 | 3.54E-06 | 0.000345 | -0.36499 | -6.08379 | 3.29E-06 | 0.000129 | TRUE | TRUE |
| LINE-1 | chr2:240141639-240142163 | 0.031287 | 5.253901 | 3.86E-06 | 0.000365 | 0.054194 | 3.438376 | 0.002341 | 0.013459 | FALSE | TRUE |
| LINE-1 | chr8:142235428-142235598 | -0.03149 | -5.16296 | 3.99E-06 | 0.000368 | -0.17988 | -2.36822 | 0.026637 | 0.062003 | FALSE | TRUE |
| LINE-1 | chr7:157594780-157595648 | -0.02225 | -5.12606 | 4.54E-06 | 0.000403 | -0.38518 | -5.12817 | 4.40E-05 | 0.000691 | TRUE | TRUE |
| LINE-1 | chr6:87607436-87613456 | -0.05216 | -5.11799 | 4.67E-06 | 0.000403 | -0.19392 | -7.43181 | 1.46E-07 | 1.88E-05 | TRUE | TRUE |
| LINE-1 | chr11:2499185-2499531 | -0.04573 | -5.11484 | 4.72E-06 | 0.000403 | -0.40786 | -5.37669 | 1.83E-05 | 0.000367 | TRUE | TRUE |
| LINE-1 | chr7:93216215-93221079 | -0.03405 | -5.10952 | 4.81E-06 | 0.000403 | -0.1189 | -3.5855 | 0.001561 | 0.010089 | FALSE | TRUE |
| LINE-1 | chr2:154107988-154113991 | -0.06516 | -5.08395 | 5.26E-06 | 0.000412 | -0.16145 | -7.8654 | 2.12E-07 | 2.20E-05 | TRUE | TRUE |
| LINE-1 | chr6:32028716-32028906 | -0.06582 | -5.07065 | 5.51E-06 | 0.000422 | -0.15336 | -1.83066 | 0.080112 | 0.131279 | FALSE | TRUE |
| LINE-1 | chr6:32192826-32193303 | -0.05905 | -5.01014 | 6.81E-06 | 0.0005 | -0.28455 | -3.2378 | 0.003725 | 0.017784 | FALSE | TRUE |
| LINE-1 | chr10:134764877-134765144 | -0.03374 | -4.98906 | 7.32E-06 | 0.000517 | -0.27141 | -3.72751 | 0.001101 | 0.007899 | FALSE | TRUE |
| LINE-1 | chr14:40817263-40823313 | -0.05322 | -4.97644 | 7.65E-06 | 0.00053 | -0.12284 | -4.01254 | 0.000583 | 0.004952 | FALSE | TRUE |
| **Supplemental Table S1** (continued) | | | | | | | | | | | |
| **RE type** | **RE location (hg19)** | **UFSH** | | | | **TCGA** | | | | **Validated^1^** | **Directionally  consistent^2^** |
|  |  | **Estimate** | **t** | **p-value** | **FDR** | **Estimate** | **t** | **p-value** | **FDR** |  |  |
| LINE-1 | chr8:143325543-143326122 | -0.09317 | -5.03084 | 8.55E-06 | 0.000581 | -0.2744 | -3.93054 | 0.000894 | 0.00687 | FALSE | TRUE |
| LINE-1 | chr1:245727087-245727152 | -0.07374 | -4.94582 | 8.83E-06 | 0.000589 | -0.31422 | -3.36377 | 0.002797 | 0.015243 | FALSE | TRUE |
| LINE-1 | chr5:8084329-8090357 | -0.06195 | -4.89748 | 1.01E-05 | 0.000659 | -0.16814 | -5.59933 | 1.47E-05 | 0.000327 | TRUE | TRUE |
| LINE-1 | chr7:44793144-44793695 | 0.040773 | 4.88986 | 1.11E-05 | 0.000667 | 0.067222 | 2.941094 | 0.007789 | 0.028227 | FALSE | TRUE |
| LINE-1 | chr8:144412153-144412234 | 0.01651 | 4.866597 | 1.12E-05 | 0.000667 | 0.016322 | 1.343247 | 0.192269 | 0.258442 | FALSE | TRUE |
| LINE-1 | chr17:103517-103586 | 0.011811 | 4.862974 | 1.13E-05 | 0.000667 | 0.01675 | 2.044361 | 0.052505 | 0.098102 | FALSE | TRUE |
| LINE-1 | chr4:8292190-8292360 | -0.10159 | -4.86085 | 1.14E-05 | 0.000667 | -0.26831 | -3.57712 | 0.001593 | 0.010218 | FALSE | TRUE |
| LINE-1 | chr3:116078847-116084873 | -0.05292 | -4.88034 | 1.15E-05 | 0.000667 | -0.11206 | -6.49354 | 1.44E-06 | 7.80E-05 | TRUE | TRUE |
| LINE-1 | chr12:118298394-118298513 | -0.02082 | -4.84215 | 1.22E-05 | 0.000697 | -0.33806 | -4.52827 | 0.000229 | 0.002454 | FALSE | TRUE |
| LINE-1 | chr11:2139904-2140461 | -0.07258 | -4.85347 | 1.26E-05 | 0.000697 | -0.19266 | -2.22187 | 0.036711 | 0.077269 | FALSE | TRUE |
| LINE-1 | chr1:234529024-234529072 | 0.016641 | 4.820273 | 1.36E-05 | 0.000731 | 0.009522 | 0.856326 | 0.400633 | 0.473551 | FALSE | TRUE |
| LINE-1 | chr2:242938503-242938722 | -0.06886 | -4.79139 | 1.45E-05 | 0.000769 | -0.3369 | -5.62016 | 1.18E-05 | 0.000283 | TRUE | TRUE |
| LINE-1 | chr8:142201551-142201827 | -0.03279 | -4.78196 | 1.50E-05 | 0.000774 | -0.2013 | -2.89884 | 0.008315 | 0.029449 | FALSE | TRUE |
| LINE-1 | chr18:77658592-77658800 | 0.021605 | 4.781108 | 1.50E-05 | 0.000774 | 0.049805 | 2.951486 | 0.007149 | 0.026538 | FALSE | TRUE |
| LINE-1 | chr7:1977994-1978347 | 0.025046 | 4.780373 | 1.56E-05 | 0.000775 | 0.025327 | 1.272789 | 0.215782 | 0.281177 | FALSE | TRUE |
| LINE-1 | chr13:73184385-73190425 | -0.0565 | -4.7696 | 1.57E-05 | 0.000775 | -0.11089 | -4.54251 | 0.000177 | 0.001999 | FALSE | TRUE |
| LINE-1 | chr13:46219166-46225202 | -0.06284 | -4.76105 | 1.61E-05 | 0.000775 | -0.10699 | -3.66557 | 0.001282 | 0.008816 | FALSE | TRUE |
| LINE-1 | chr5:119112282-119116494 | -0.06913 | -4.7694 | 1.62E-05 | 0.000775 | -0.14387 | -4.03659 | 0.000512 | 0.004513 | FALSE | TRUE |
| LINE-1 | chr12:133516736-133516924 | -0.0226 | -4.76939 | 1.62E-05 | 0.000775 | 0.008331 | 0.606198 | 0.550308 | 0.613343 | FALSE | FALSE |
| LINE-1 | chr7:75296217-75296292 | 0.017989 | 4.756719 | 1.64E-05 | 0.000775 | 0.024063 | 2.024271 | 0.054682 | 0.100829 | FALSE | TRUE |
| LINE-1 | chr7:157318533-157319261 | -0.06548 | -4.75627 | 1.69E-05 | 0.000792 | -0.44302 | -9.66384 | 2.19E-09 | 2.05E-06 | TRUE | TRUE |
| LINE-1 | chr2:117913611-117919627 | -0.05926 | -4.71594 | 1.88E-05 | 0.000858 | -0.15001 | -5.4004 | 1.99E-05 | 0.00039 | TRUE | TRUE |
| LINE-1 | chr6:102617742-102623571 | -0.06224 | -4.70094 | 1.98E-05 | 0.000876 | -0.16164 | -8.35541 | 1.99E-08 | 5.07E-06 | TRUE | TRUE |
| LINE-1 | chr11:133928347-133928612 | -0.1064 | -4.6917 | 2.04E-05 | 0.000876 | -0.25821 | -3.21622 | 0.003969 | 0.018541 | FALSE | TRUE |
| LINE-1 | chr12:21572150-21578168 | -0.07052 | -4.69163 | 2.04E-05 | 0.000876 | -0.13597 | -4.55642 | 0.000154 | 0.001822 | FALSE | TRUE |
| LINE-1 | chr8:142852678-142853054 | -0.0596 | -4.6884 | 2.07E-05 | 0.000876 | -0.4085 | -5.17699 | 2.99E-05 | 0.000522 | TRUE | TRUE |
| LINE-1 | chr8:143458657-143458848 | 0.035875 | 4.728466 | 2.14E-05 | 0.000891 | 0.106543 | 5.479939 | 2.73E-05 | 0.000486 | TRUE | TRUE |
| LINE-1 | chr10:64362510-64368514 | -0.05871 | -4.67638 | 2.15E-05 | 0.000891 | -0.09164 | -3.62651 | 0.001464 | 0.009616 | FALSE | TRUE |
| LINE-1 | chr12:124751387-124751850 | -0.02939 | -4.66896 | 2.21E-05 | 0.000895 | -0.37849 | -4.85408 | 7.20E-05 | 0.001017 | FALSE | TRUE |
| LINE-1 | chr5:511306-512394 | -0.02949 | -4.67776 | 2.21E-05 | 0.000895 | -0.27512 | -3.50748 | 0.001952 | 0.011911 | FALSE | TRUE |
| LINE-1 | chr2:1166233-1166668 | -0.07127 | -4.70007 | 2.27E-05 | 0.000908 | -0.27632 | -5.77468 | 1.44E-05 | 0.000326 | TRUE | TRUE |
| LINE-1 | chr16:1160593-1160798 | -0.02204 | -4.62804 | 2.54E-05 | 0.000994 | -0.14788 | -1.52039 | 0.142013 | 0.203745 | FALSE | TRUE |
| LINE-1 | chr7:124819625-124822032 | -0.05599 | -4.63219 | 2.58E-05 | 0.001 | -0.23331 | -5.00904 | 4.93E-05 | 0.000757 | TRUE | TRUE |
| Alu | chr19:54253716-54253980 | -0.0727 | -6.22973 | 5.40E-08 | 0.000258 | -0.30737 | -4.47691 | 0.000115 | 0.014738 | FALSE | TRUE |
| Alu | chr15:99563481-99563734 | -0.08867 | -6.14091 | 8.51E-08 | 0.000279 | -0.21253 | -4.52985 | 0.000115 | 0.014738 | FALSE | TRUE |
| Alu | chr13:30062564-30062769 | -0.04699 | -6.09237 | 9.15E-08 | 0.000279 | -0.27507 | -6.42106 | 9.85E-07 | 0.000478 | TRUE | TRUE |
| Alu | chrX:103294139-103294294 | -0.04791 | -5.89744 | 1.93E-07 | 0.000433 | -0.38972 | -6.23305 | 1.13E-06 | 0.000519 | TRUE | TRUE |
| Alu | chr6:31598686-31598816 | 0.092924 | 5.627963 | 5.34E-07 | 0.000849 | 0.137329 | 5.776961 | 2.90E-06 | 0.000955 | TRUE | TRUE |
| Alu | chr6:127534521-127534806 | -0.04194 | -5.55913 | 8.65E-07 | 0.000952 | -0.03816 | -1.62979 | 0.113918 | 0.521021 | FALSE | TRUE |
| Alu | chr11:989047-989352 | 0.044711 | 5.50402 | 1.01E-06 | 0.000991 | 0.012873 | 0.453796 | 0.65334 | 0.80339 | FALSE | TRUE |

^1^ REs with FDR <0.001 using both of our testing dataset (UFSH) and validation dataset (TCGA) are validated REs.

^2^ REs with the same direction of the estimates are directionally consistent. Compared to normal liver, negative estimates indicate hypomethylation in HCV-HCC and positive estimates indicate hypermethylation in HCV-HCC.

Table S3. **Differentially methylated LINE-1 and Alu in HCV-HCC (FDR <0.001) that were directionally consistent in HCV-cirrhosis.**

| **RE type** | **RE location (hg19)^1^** | **Proximal gene symbol** | **HCV-HCC (UFSH)** | | | | **HCV-cirrhosis** | | | | **HCV-HCC (TCGA)^1^** | | | |
| --- | --- | --- | --- | --- | --- | --- | --- | --- | --- | --- | --- | --- | --- | --- |
|  |  |  | **Estimate** | **t** | **p-value** | **FDR** | **Estimate** | **t** | **p-value** | **FDR** | **Estimate** | **t** | **p-value** | **FDR** |
| LINE-1 | chr7:5642119-5642313 | *FSCN1* | -0.0255 | -4.9081 | 1.13E-05 | 0.0007 | -0.0145 | -3.0607 | 0.0036 | 0.0879 | NA | NA | NA | NA |
| LINE-1 | chr11:67350137-67350490 | *GSTP1* | 0.1236 | 4.7633 | 1.66E-05 | 0.0008 | 0.1041 | 3.7517 | 0.0005 | 0.0284 | 0.1203 | 2.6679 | 0.0139 | 0.0407 |
| LINE-1 | chr11:133928347-133928612 | *JAM3*  (upstream)^2^ | -0.1064 | -4.6917 | 2.04E-05 | 0.0009 | -0.0752 | -3.2760 | 0.0019 | 0.0655 | -0.2582 | -3.2162 | 0.0040 | 0.0185 |
| Alu | chr8:42608585-42608842 | *CHRNA6* | -0.0720 | -6.4773 | 2.93E-08 | 0.0002 | -0.0378 | -3.8113 | 0.0004 | 0.0827 | NA | NA | NA | NA |
| Alu | chr16:69565395-69565686 | *NFAT5*  (upstream)^3^ | -0.0860 | -6.2502 | 9.02E-08 | 0.0003 | -0.0592 | -3.7107 | 0.0005 | 0.0941 | NA | NA | NA | NA |
| Alu | chr6:31598686-31598816 | *PRRC2A* | 0.0929 | 5.6280 | 5.34E-07 | 0.0008 | 0.0658 | 3.7094 | 0.0005 | 0.0921 | 0.1373 | 5.7770 | 2.90E-06 | 0.0010 |

^1^ Methylation data of 3 REs were not available in TCGA.

^2^ Intergenic LINE-1 located upstream of nearest gene *JAM3* with distance of 10208 bp.

^3^ Intergenic Alu located upstream of nearest gene *NFAT5* with distance of 34183 bp.

Table S4. **Enrichment of the 15 HCV-HCC REs in four regulatory histone modification marks measured in normal liver tissue (ID: E066) in Roadmap Epigenomics Project**

| **Histone modification marker** | **Observed count^1^** | **Expected count^2^** | | **p-value^3^** | **Direction** |
| --- | --- | --- | --- | --- | --- |
|  |  | **Mean** | **Standard deviation** |  |  |
| H3K4me1 | 3 | 3.50 | 1.61 | 0.379 | Depleted |
| H3K4me3 | 2 | 3.18 | 1.56 | 0.225 | Depleted |
| H3K27ac | 0 | 2.68 | 1.50 | 0.037 | Depleted |
| H3K27me3 | 6 | 3.10 | 1.55 | 0.030 | Enriched |

^1^ Count of REs that overlapped with the histone markers.

^2^ Distribution of the count of matched and randomly selected REs (10000 null sets) that overlapped with the histone markers.

^3^ Empirical p-value calculated by comparing the distribution of null count and the observed count.

Table S5. **Coefficients of HCV-HCC RE methylation score^1^**

|  | **Coefficient (weight)**^2^ | **RE location (hg19)** | **Proximal gene symbol** | **Hypomethylated?**^3^ |
| --- | --- | --- | --- | --- |
| (Intercept) | 1.921280462 |  |  |  |
| LINE-1 | 0 | chr10:134875427-134876211 | *ADGRA1* | Yes |
| LINE-1 | 0.428113711 | chr6:9810984-9817010 | *TFAP2A* | Yes |
| LINE-1 | 0 | chr7:4388884-4389235 | *SDK1* | Yes |
| LINE-1 | 0 | chr7:4085177-4085587 | *SDK1* | Yes |
| LINE-1 | 0 | chr7:157594780-157595648 | *PTPRN2* | Yes |
| LINE-1 | 0 | chr11:2499185-2499531 | *KCNQ1* | Yes |
| LINE-1 | 0 | chr5:8084329-8090357 | *MTRR* | Yes |
| LINE-1 | 0 | chr2:242938503-242938722 | *LINC01237* | Yes |
| LINE-1 | 0 | chr7:157318533-157319261 | *PTPRN2* | Yes |
| LINE-1 | 0 | chr8:142852678-142853054 | *MROH5* | Yes |
| LINE-1 | -0.131762135 | chr8:143458657-143458848 | *TSNARE1* | No |
| LINE-1 | 0.008153261 | chr2:1166233-1166668 | *SNTG2* | Yes |
| Alu | 0.685384507 | chr13:30062564-30062769 | *MTUS2* | Yes |
| Alu | 0.305877155 | chrX:103294139-103294294 | *H2BFM* | Yes |
| Alu | -0.687902233 | chr6:31598686-31598816 | *PRRC2A* | No |

^1^ Presented as the same order as Table 2.

^2^ Zero coefficients indicate the REs were not selected (i.e. zero contribution) in the HCV-HCC RE methylation score. The score was computed as a weighted sum of methylation level of these REs using their coefficients as weight.

^3^ Positive coefficients were assigned to hypomethylated RE while negative coefficients were assigned to hypermethylated REs.

SUPPLEMENTARY REFERENCES

1. Aryee MJ, Jaffe AE, Corrada-Bravo H, Ladd-Acosta C, Feinberg AP, Hansen KD, Irizarry RA. Minfi: a flexible and comprehensive Bioconductor package for the analysis of Infinium DNA methylation microarrays. Bioinformatics 2014; 30:1363-9.

2. Xu Z, Niu L, Li L, Taylor JA. ENmix: a novel background correction method for Illumina HumanMethylation450 BeadChip. Nucleic acids research 2016; 44:e20.

3. Troyanskaya O, Cantor M, Sherlock G, Brown P, Hastie T, Tibshirani R, Botstein D, Altman RB. Missing value estimation methods for DNA microarrays. Bioinformatics 2001; 17:520-5.

4. Love MI, Huber W, Anders S. Moderated estimation of fold change and dispersion for RNA-seq data with DESeq2. Genome Biol 2014; 15:550.

5. Hlady RA, Sathyanarayan A, Thompson JJ, Zhou D, Wu Q, Pham K, Lee JH, Liu C, Robertson KD. Integrating the Epigenome to Identify Novel Drivers of Hepatocellular Carcinoma. Hepatology 2018.

6. Friedman J, Hastie T, Tibshirani R. Regularization Paths for Generalized Linear Models via Coordinate Descent. J Stat Softw 2010; 33:1-22.
