# Supplementary material for: DNA methylation of individual repetitive elements in hepatitis C virus infection-induced hepatocellular carcinoma
Source: Clin Epigenetics. 2019 Oct 21;11:145. doi: 10.1186/s13148-019-0733-y (PMC6802191; doi:10.1186/s13148-019-0733-y)

Extended Figures

Zheng et. al.
DNA Methylation of Individual Repetitive Elements in Hepatitis C Virus Infection-Induced Hepatocellular Carcinoma

Note: The following figures provide the genomic views of the identified 15 HCV-HCC-associated RE and their proximal gene within 500 kb (one gene per figure; presented as the same order as Table 2). Mean methylation levels for each CpGs in REs across HCV-HCC and normal liver tissue are plotted to demonstrate the hypo-/hyper-methylation patterns. The identified differentially methylated RE region(s) are highlighted by red shaded frame(s). Both UFSH and TCGA results are shown.

LINE-1 chr10:134875427-134876211

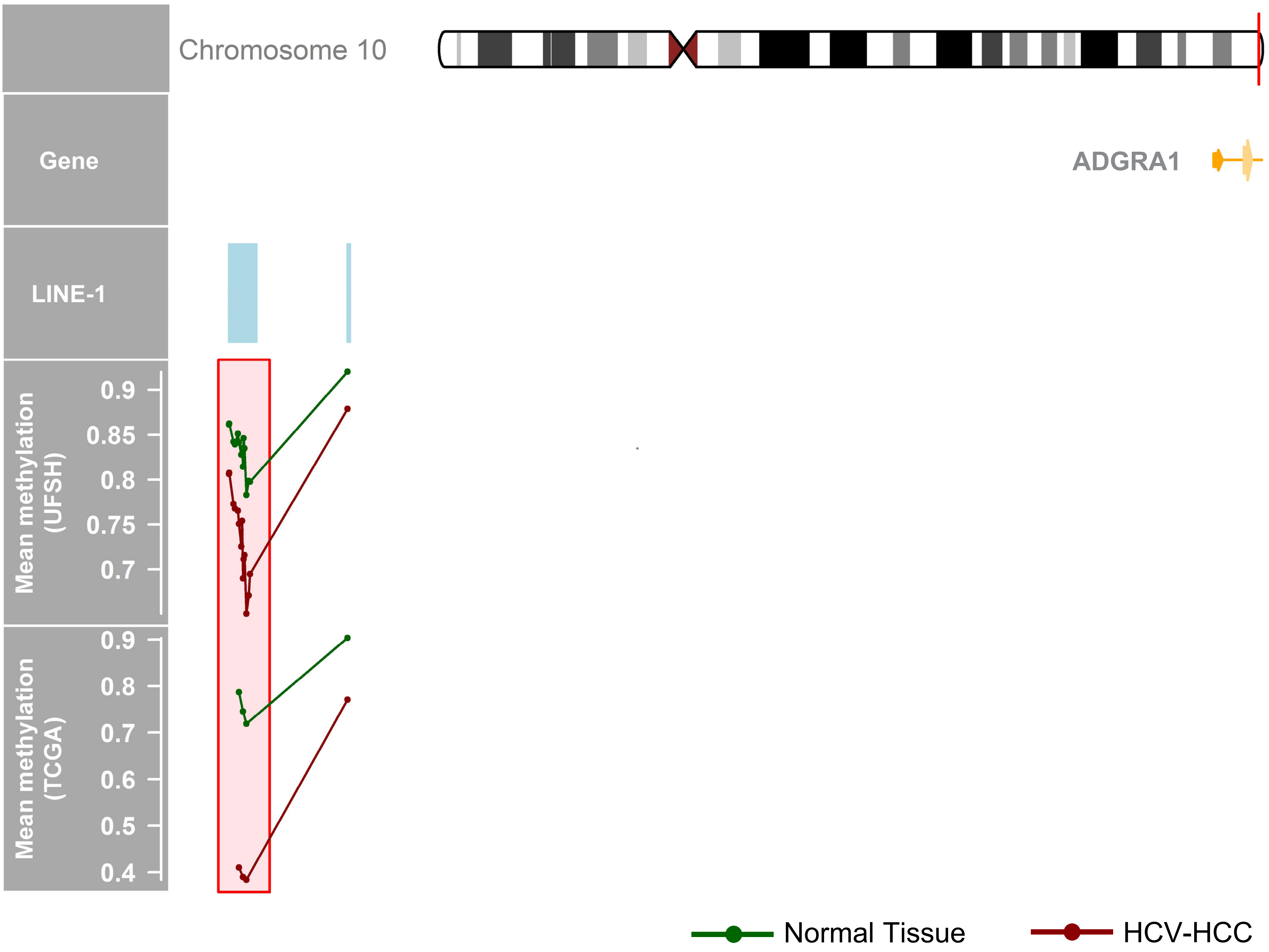

LINE-1 chr6:9810984-9817010

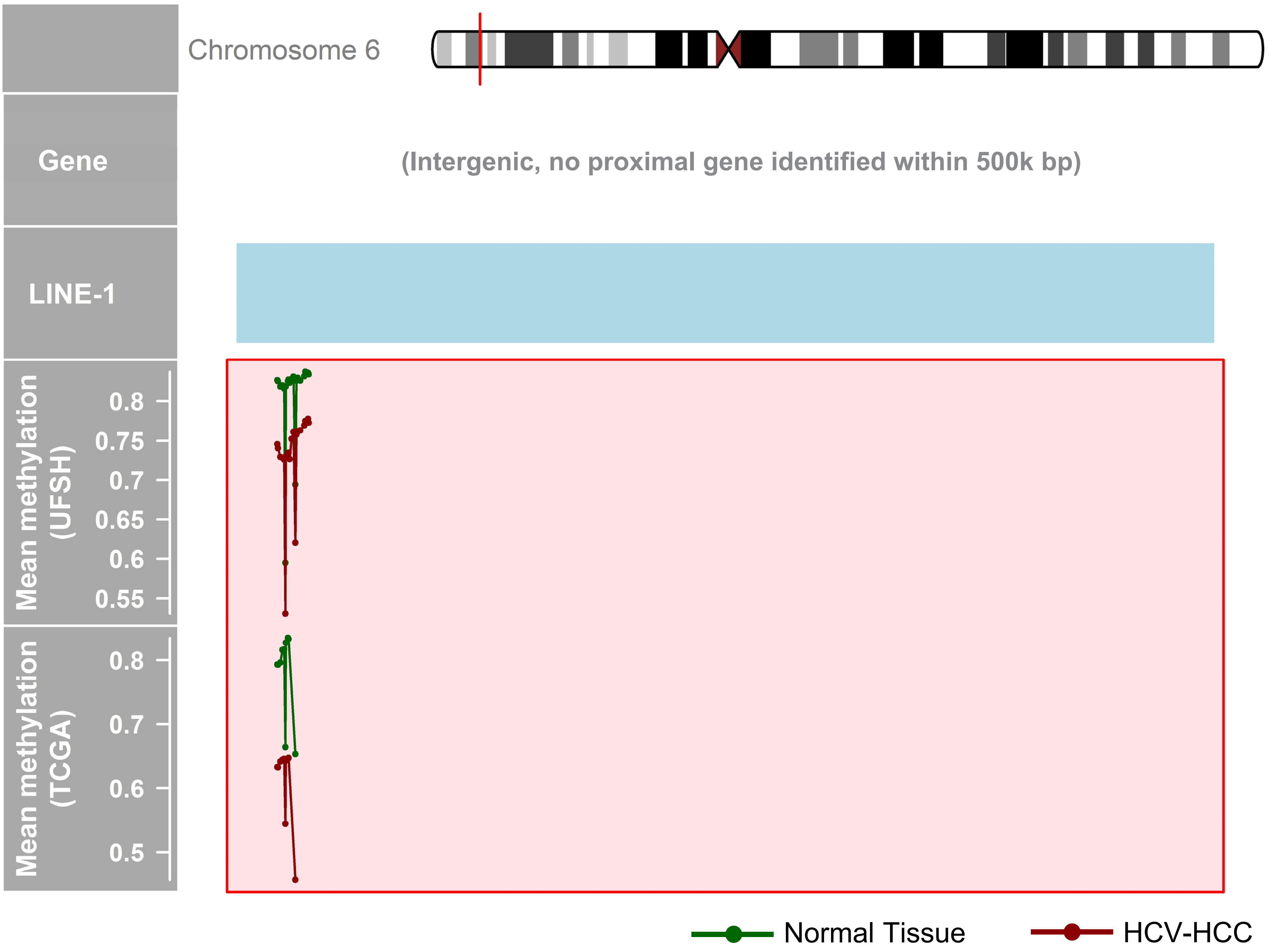

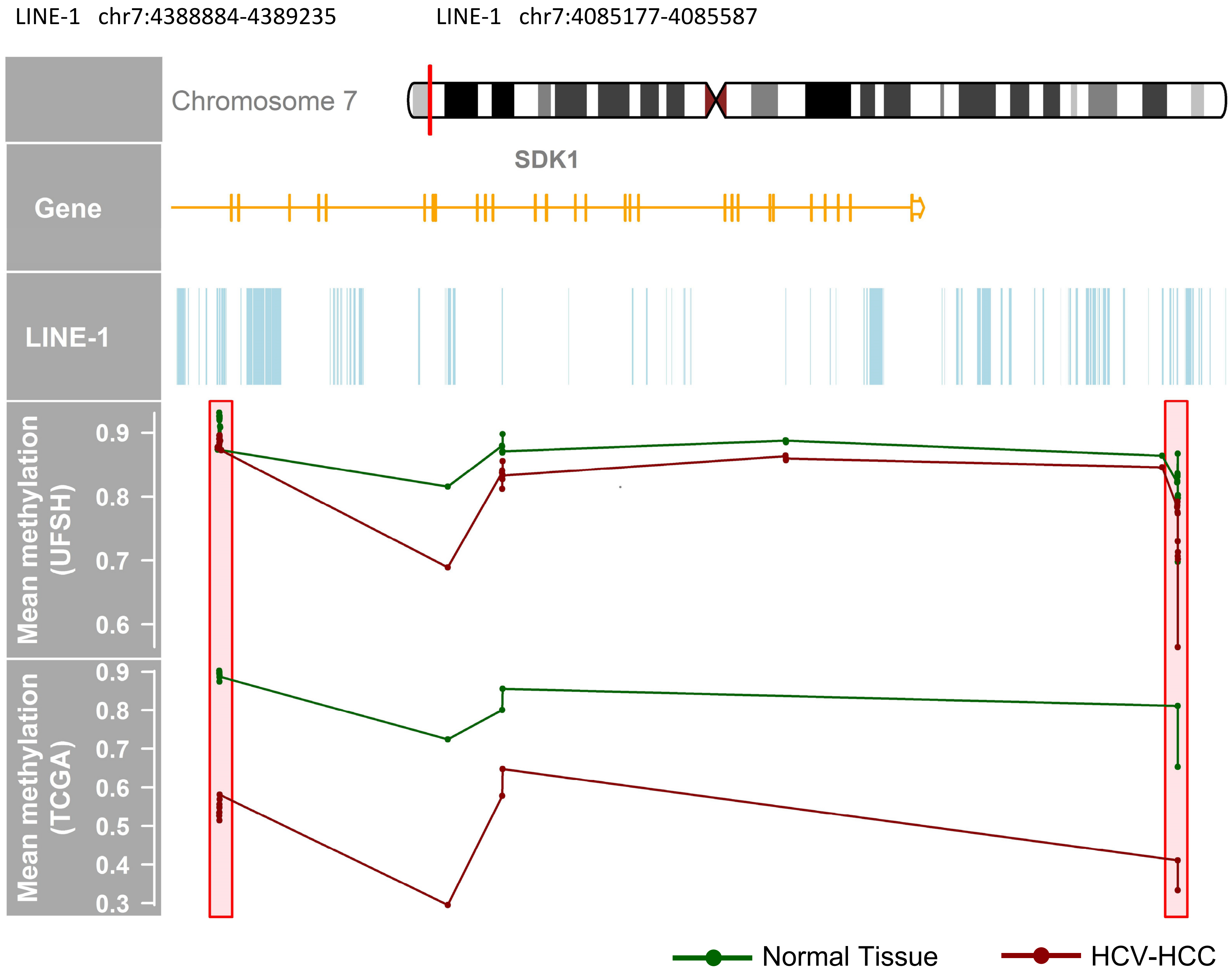

LINE-1 chr7:4388884-4389235
LINE-1 chr7:4085177-4085587
Chromosome 7
SDK1
Gene
LINE-1
Mean methylation (UFSH)
0.9
0.8
0.7
0.6
Mean methylation (TCGA)
0.9
0.8
0.7
0.6
0.5
0.4
0.3
Normal Tissue
HCV-HCC

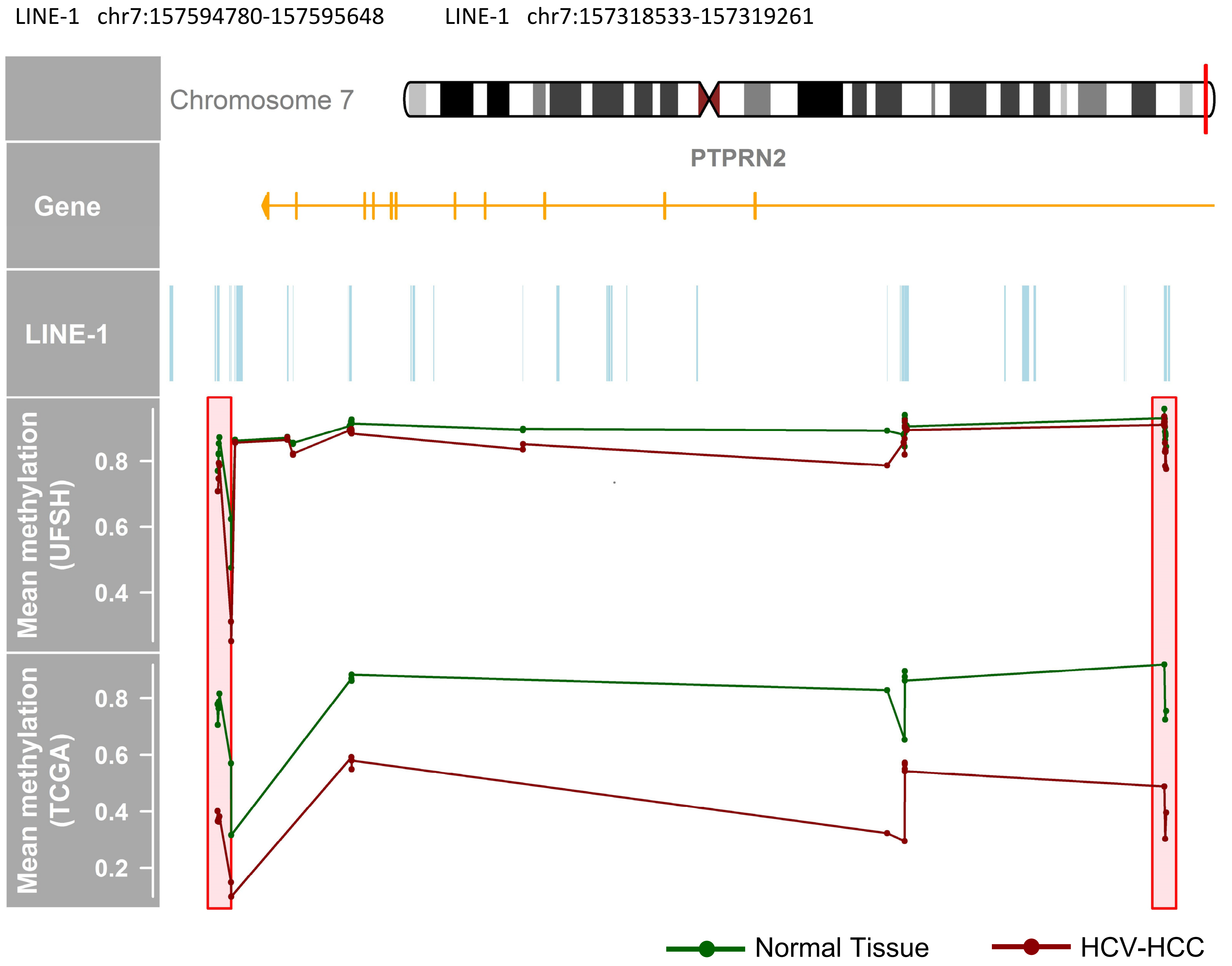

LINE-1 chr7:157594780-157595648
LINE-1 chr7:157318533-157319261
Chromosome 7
Gene
PTPRN2
LINE-1
Mean methylation (UFSH)
0.8
0.6
0.4
Mean methylation (TCGA)
0.8
0.6
0.4
0.2
Normal Tissue
HCV-HCC

LINE-1 chr11:2499185-2499531

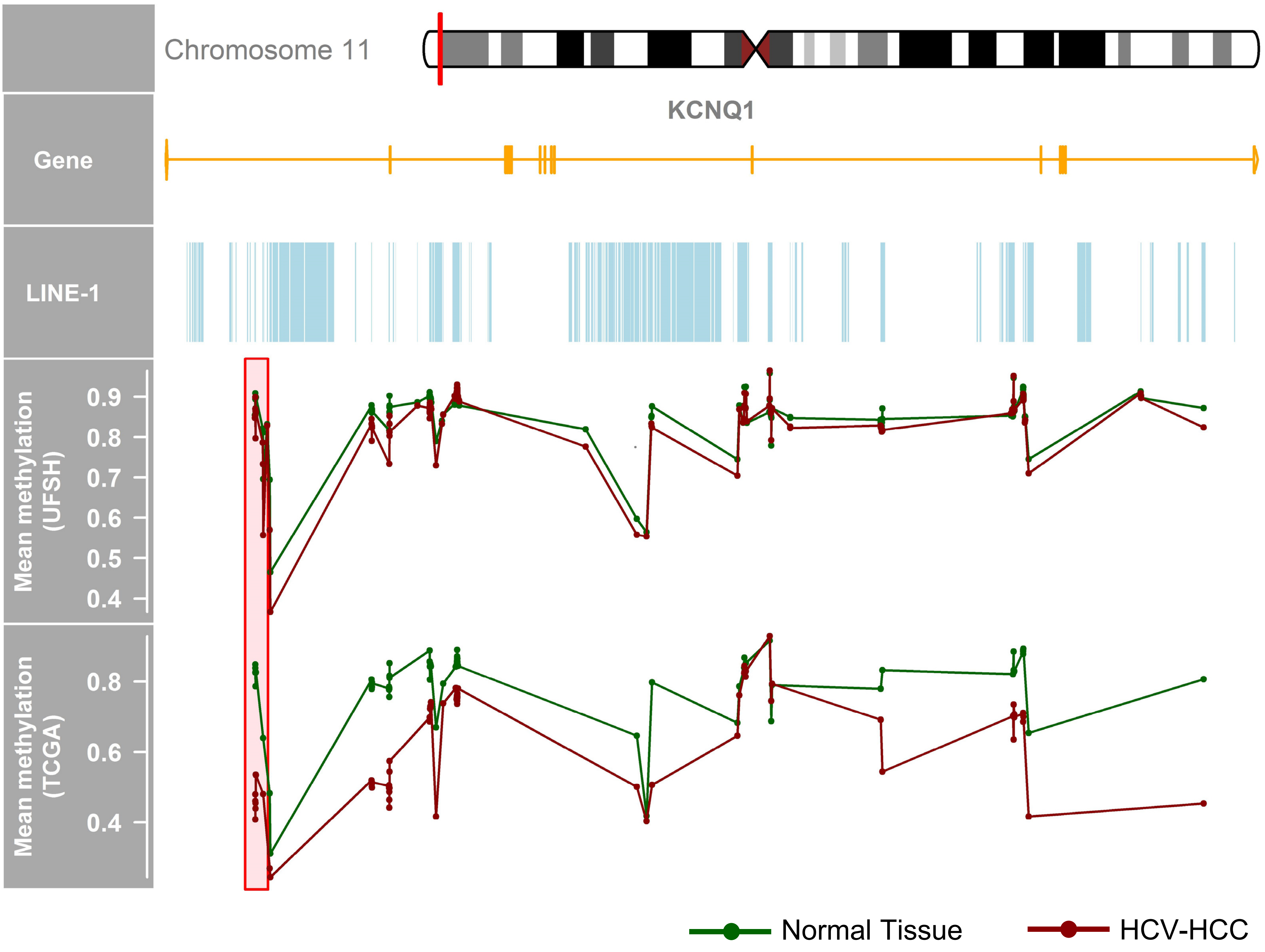

LINE-1 chr5:8084329-8090357

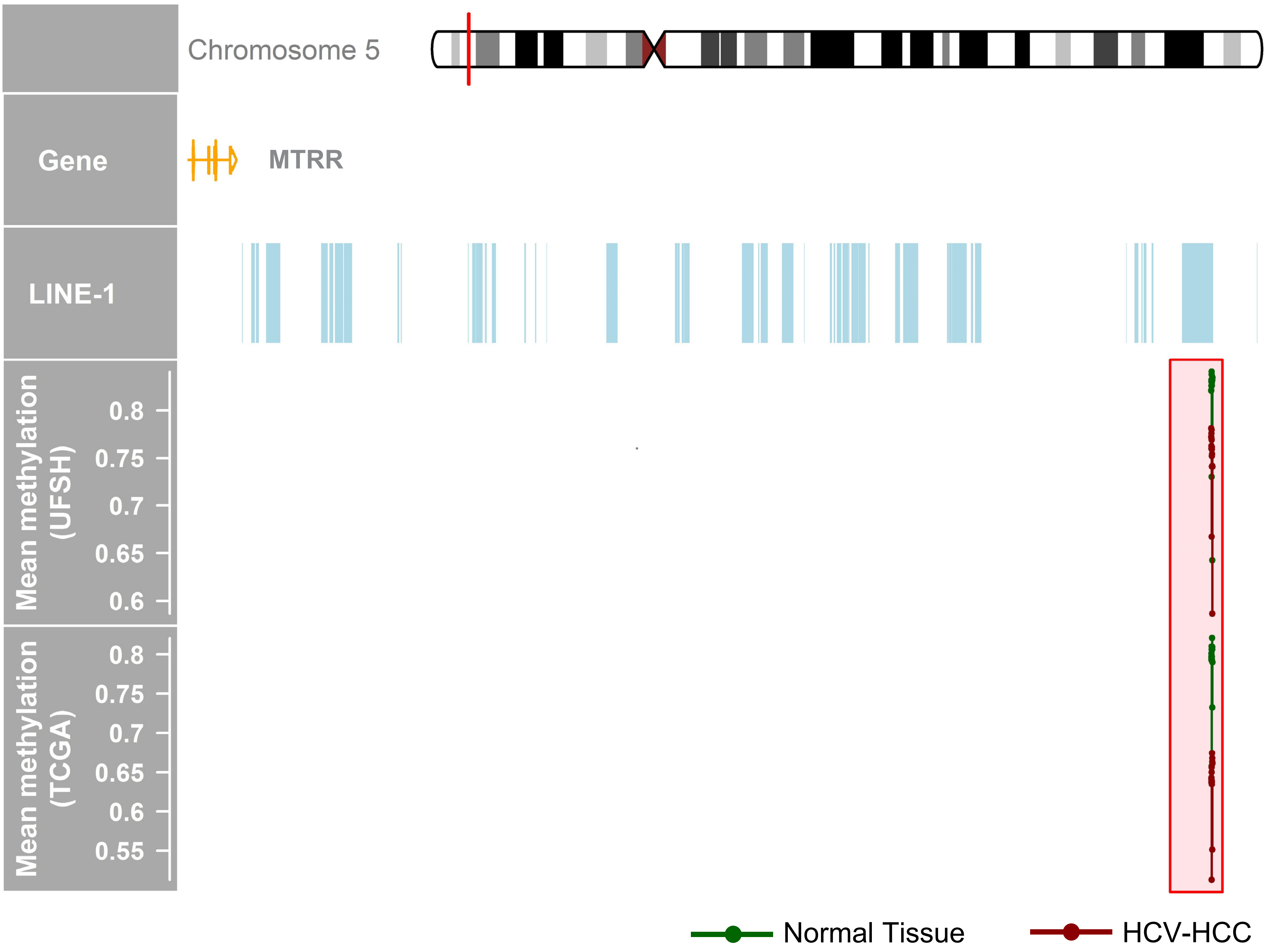

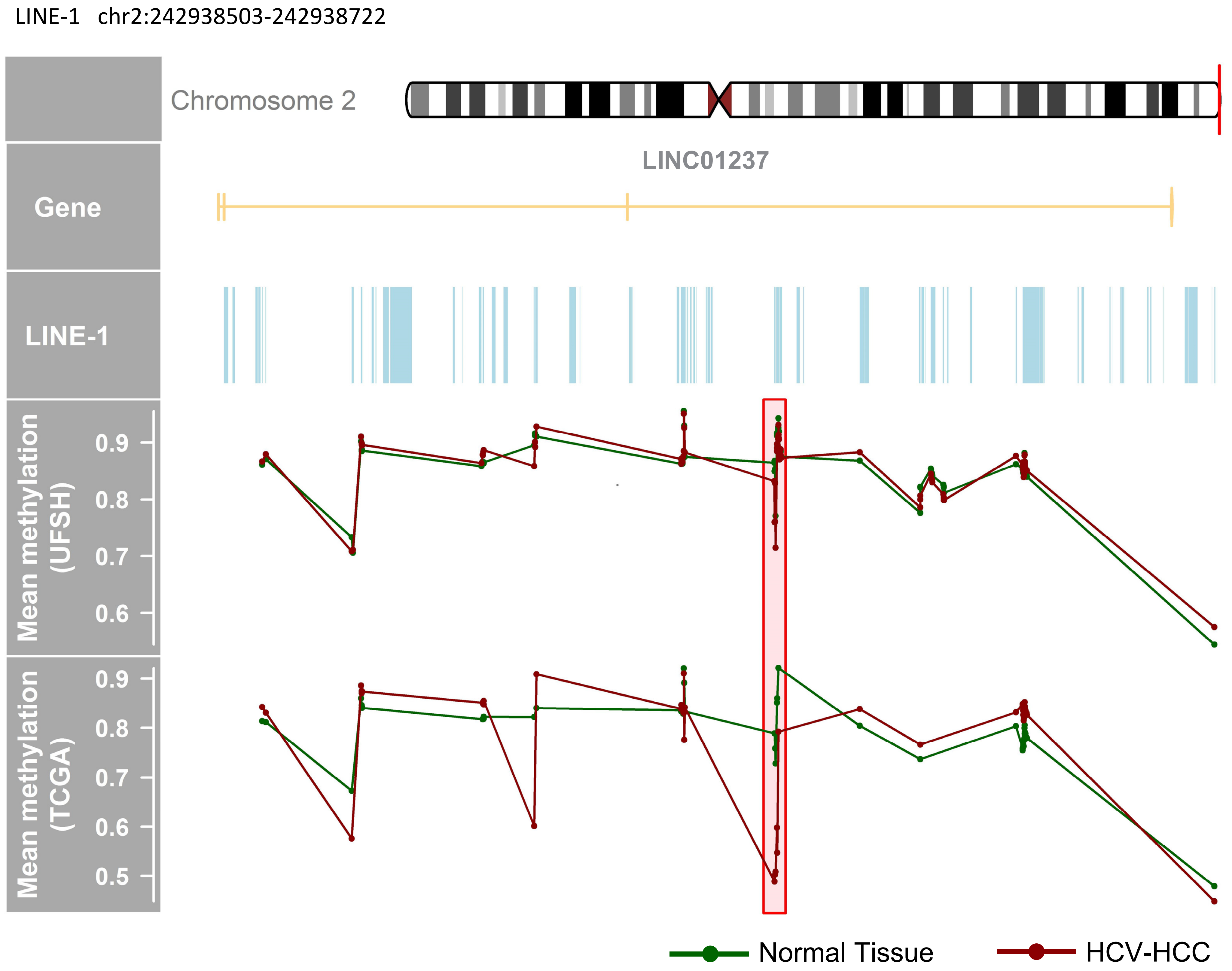

LINE-1 chr2:242938503-242938722
Chromosome 2
Gene
LINC01237
LINE-1
Mean methylation (UFSH)
0.9
0.8
0.7
0.6
Mean methylation (TCGA)
0.9
0.8
0.7
0.6
0.5
Normal Tissue
HCV-HCC

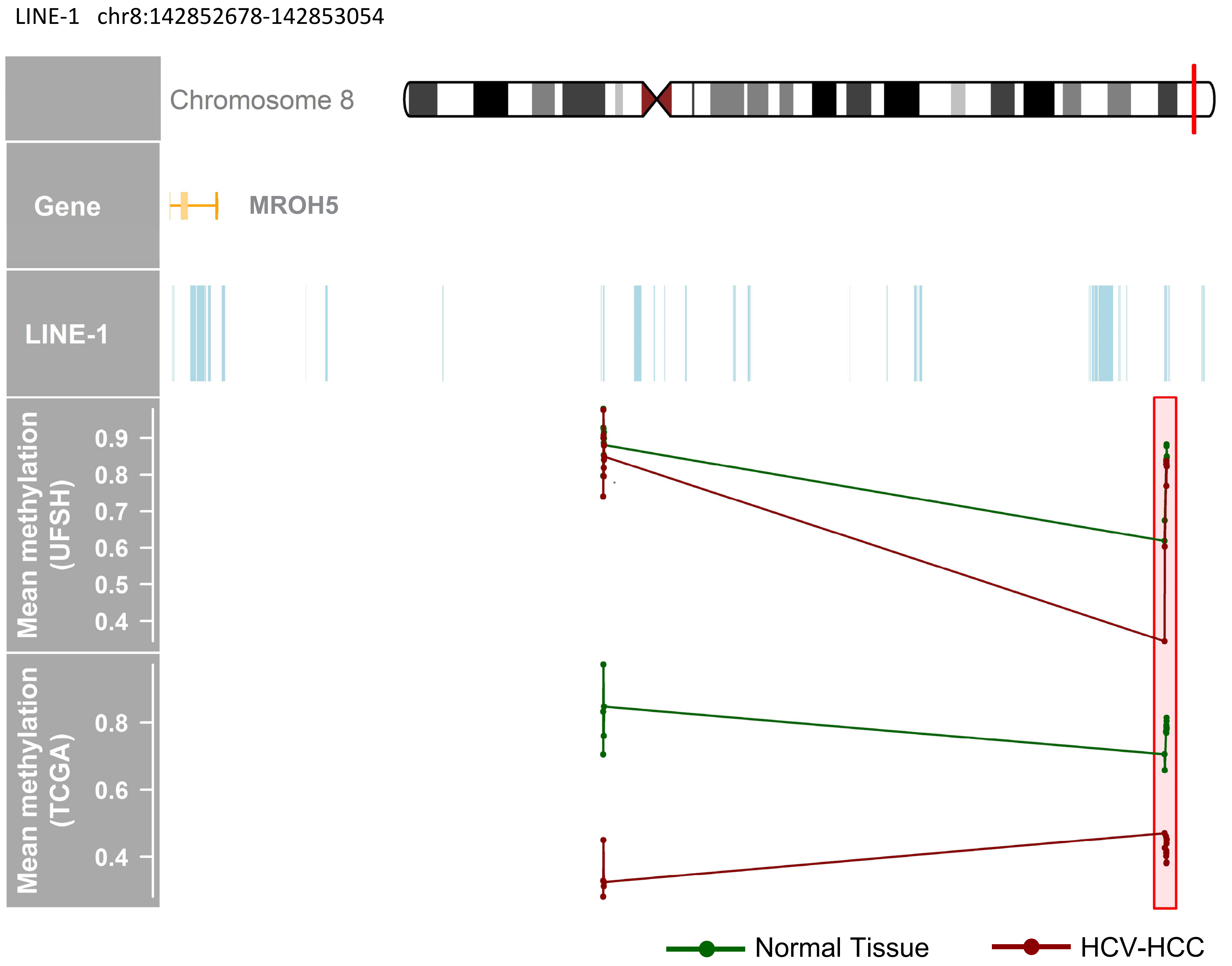

LINE-1 chr8:142852678-142853054
Chromosome 8
Gene
MROH5
LINE-1
Mean methylation (UFSH)
0.9
0.8
0.7
0.6
0.5
0.4
Mean methylation (TCGA)
0.8
0.6
0.4
Normal Tissue
HCV-HCC

LINE-1 chr8:143458657-143458848

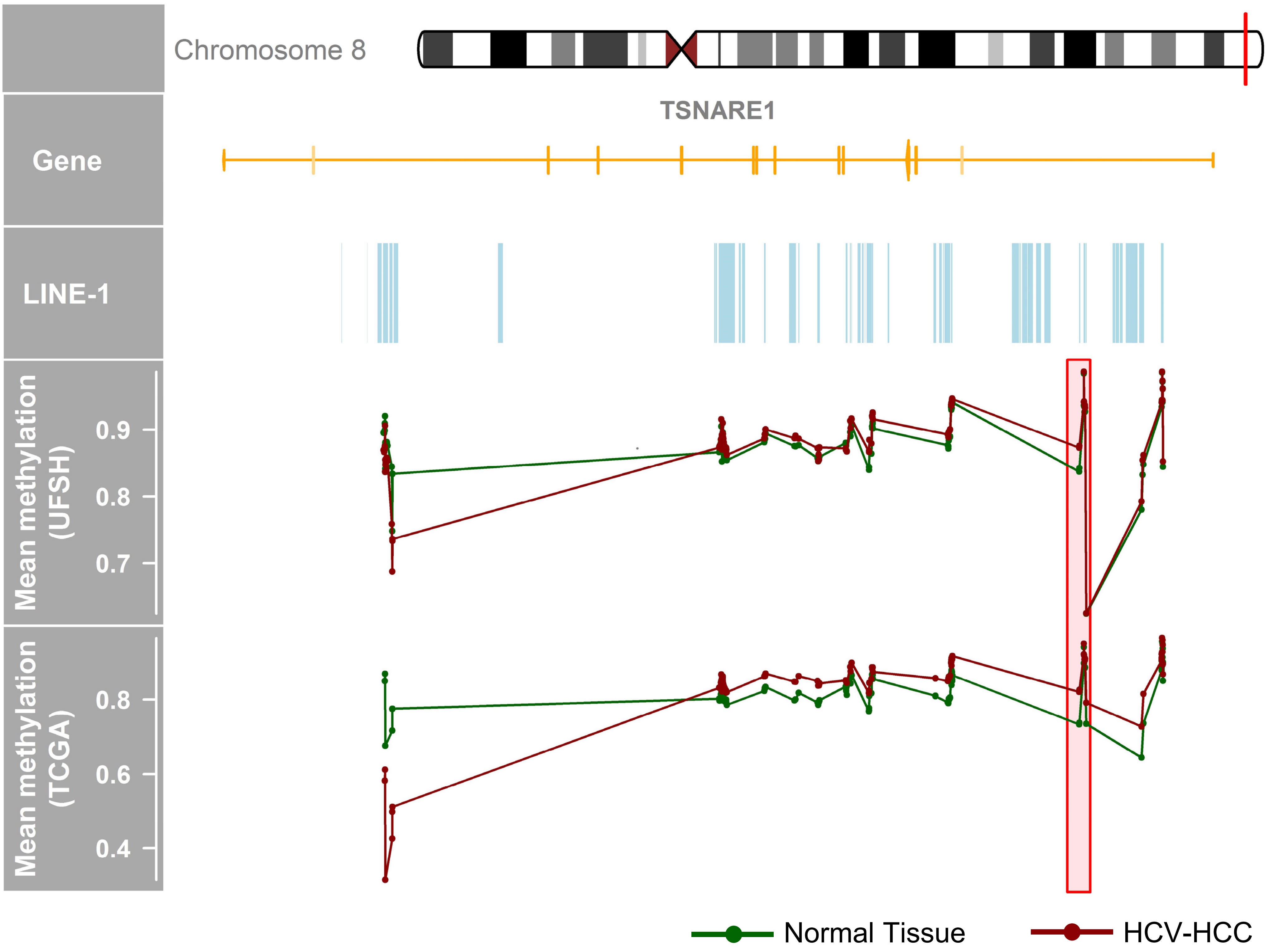

LINE-1 chr2:1166233-1166668

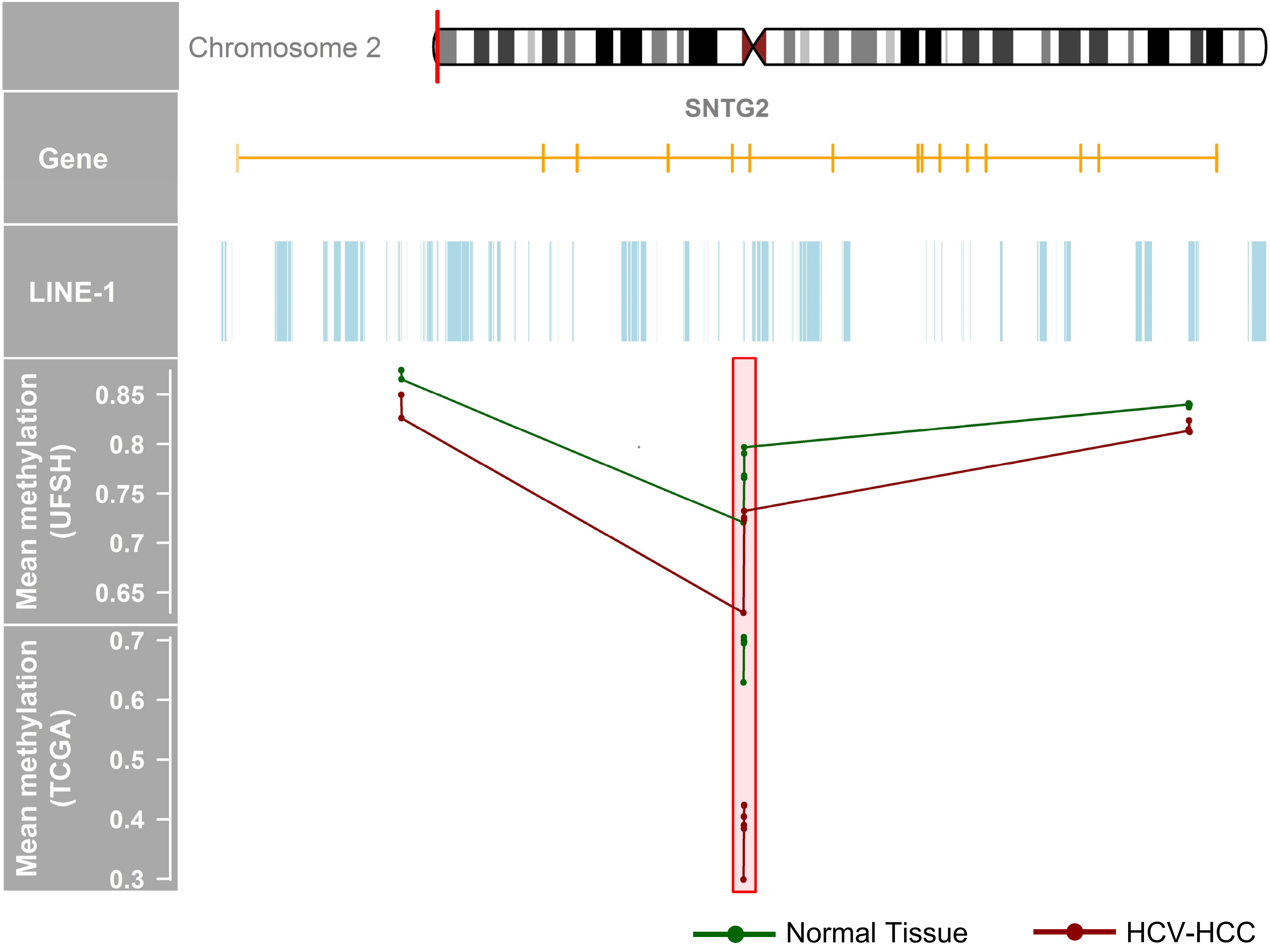

Alu chr13:30062564-30062769

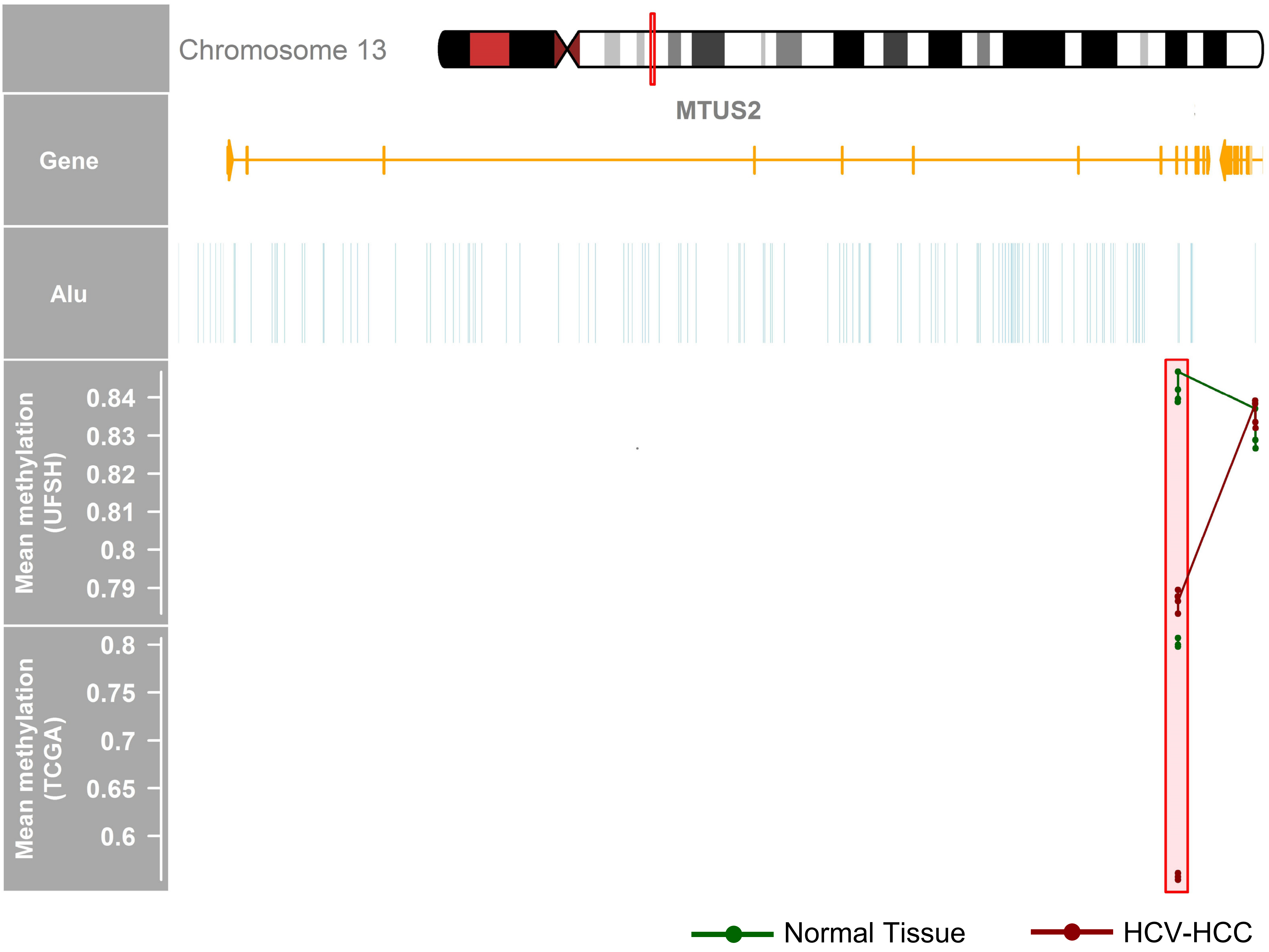

Alu chrX:103294139-103294294

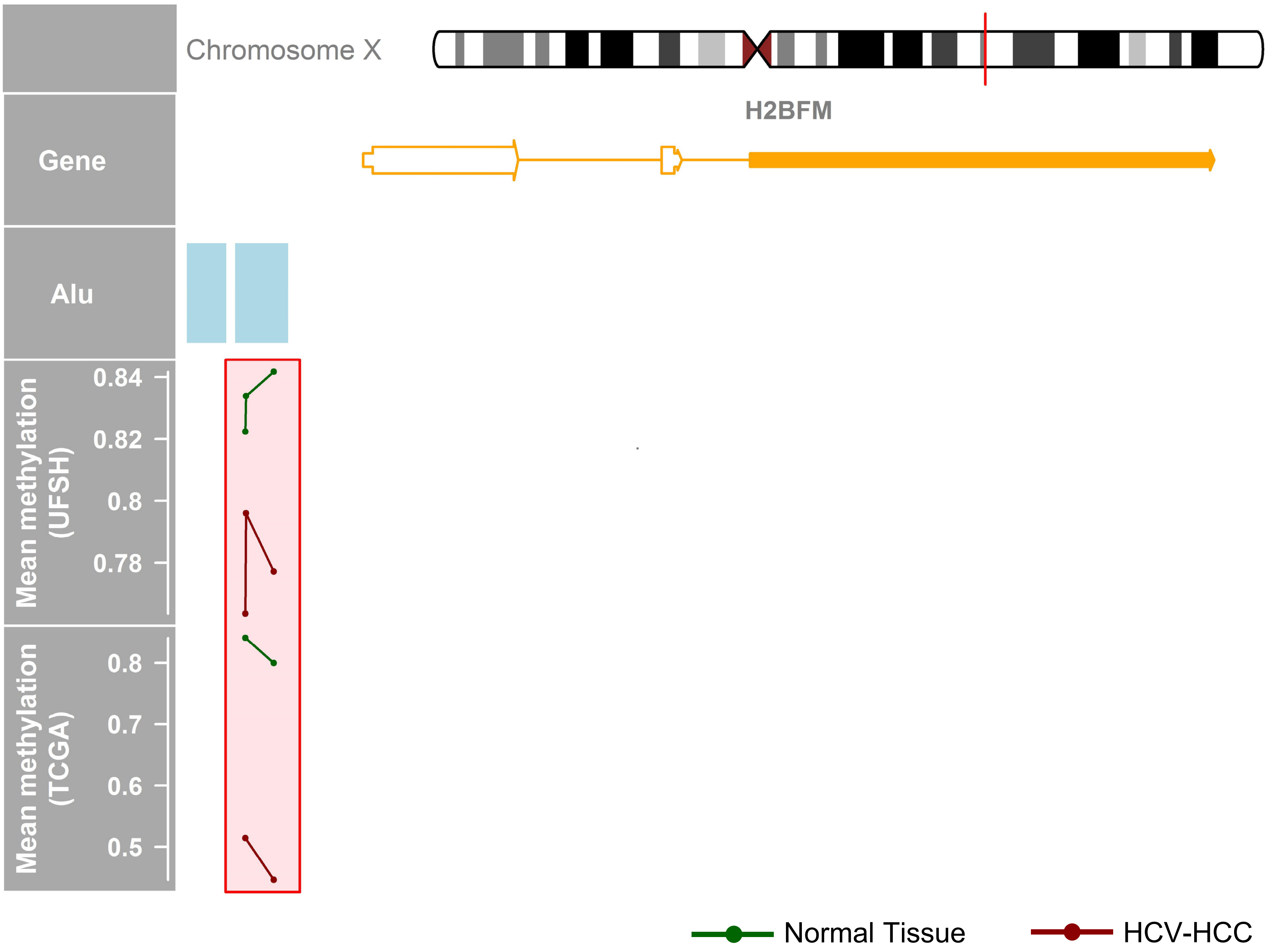

Alu chr6:31598686-31598816

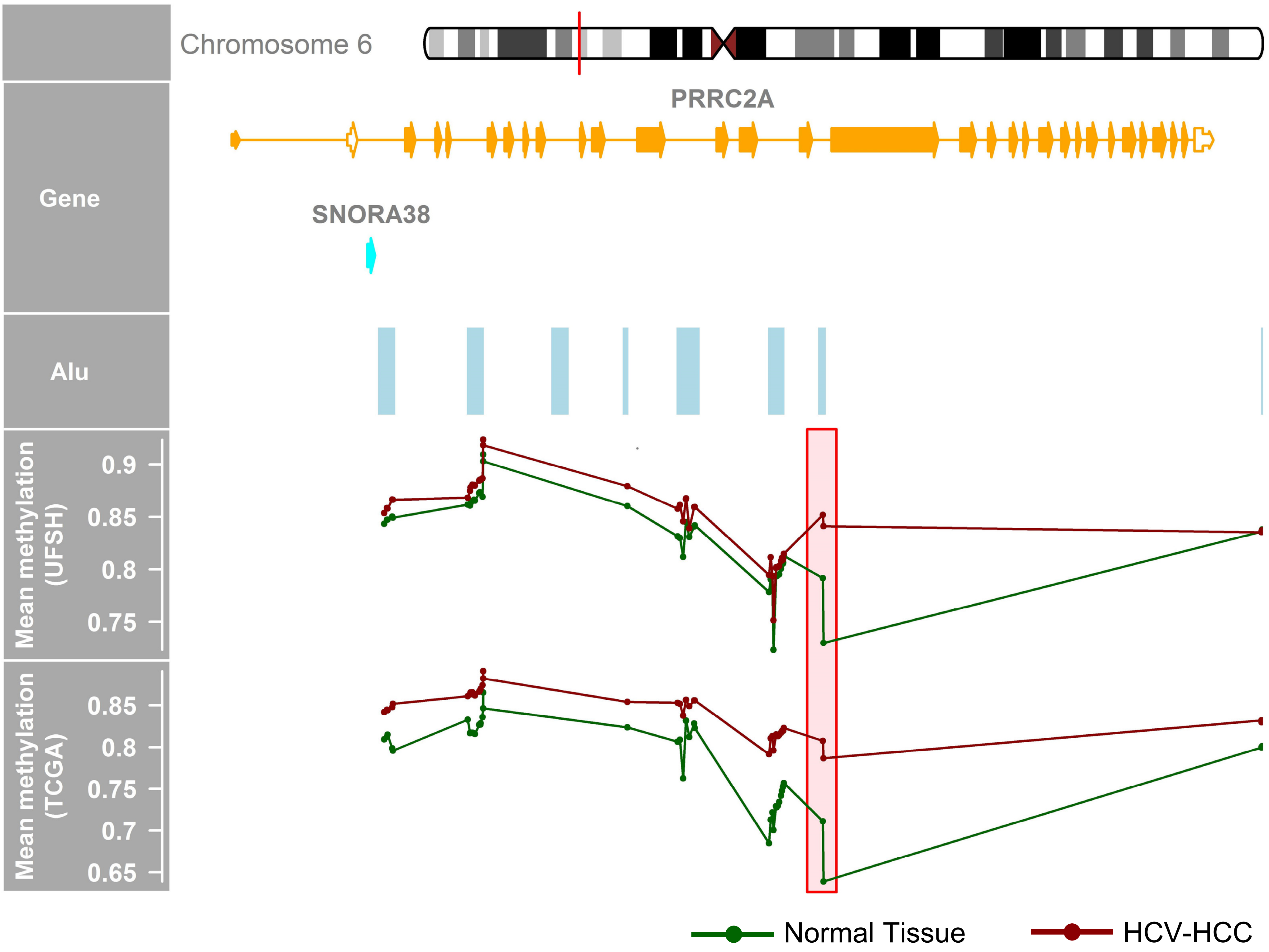

Supplement: Supplementary file 2 — Extended figures. (PDF 7145 kb) [file 13148_2019_733_MOESM2_ESM.pdf]
